# The *Drosophila* Zinc Finger Transcription Factor Ouija Board Controls Ecdysteroid Biosynthesis through Specific Regulation of *spookier*

**DOI:** 10.1371/journal.pgen.1005712

**Published:** 2015-12-10

**Authors:** Tatsuya Komura-Kawa, Keiko Hirota, Yuko Shimada-Niwa, Rieko Yamauchi, MaryJane Shimell, Tetsuro Shinoda, Akiyoshi Fukamizu, Michael B. O’Connor, Ryusuke Niwa

**Affiliations:** 1 Graduate School of Life and Environmental Sciences, University of Tsukuba, Tsukuba, Ibaraki, Japan; 2 Life Science Center, Tsukuba Advanced Research Alliance, University of Tsukuba, Tsukuba, Ibaraki, Japan; 3 Department of Genetics, Cell Biology and Development, University of Minnesota, Minneapolis, Minnesota, United States of America; 4 National Institute of Agrobiological Sciences, Tsukuba, Ibaraki, Japan; 5 PRESTO, Japan Science and Technology Agency, Kawaguchi, Saitama, Japan; K.U.Leuven, BELGIUM

## Abstract

Steroid hormones are crucial for many biological events in multicellular organisms. In insects, the principal steroid hormones are ecdysteroids, which play essential roles in regulating molting and metamorphosis. During larval and pupal development, ecdysteroids are synthesized in the prothoracic gland (PG) from dietary cholesterol via a series of hydroxylation and oxidation steps. The expression of all but one of the known ecdysteroid biosynthetic enzymes is restricted to the PG, but the transcriptional regulatory networks responsible for generating such exquisite tissue-specific regulation is only beginning to be elucidated. Here, we report identification and characterization of the C_2_H_2_-type zinc finger transcription factor Ouija board (Ouib) necessary for ecdysteroid production in the PG in the fruit fly *Drosophila melanogaster*. Expression of *ouib* is predominantly limited to the PG, and genetic null mutants of *ouib* result in larval developmental arrest that can be rescued by administrating an active ecdysteroid. Interestingly, *ouib* mutant animals exhibit a strong reduction in the expression of one ecdysteroid biosynthetic enzyme, *spookier*. Using a cell culture-based luciferase reporter assay, Ouib protein stimulates transcription of *spok* by binding to a specific ~15 bp response element in the *spok* PG enhancer element. Most remarkable, the developmental arrest phenotype of *ouib* mutants is rescued by over-expression of a functionally-equivalent paralog of *spookier*. These observations imply that the main biological function of Ouib is to specifically regulate *spookier* transcription during *Drosophila* development.

## Author Summary

Steroid hormones are crucial for development and reproduction in multicellular organisms. The spatially-restricted expression of almost all steroid biosynthesis genes is key to the specialization of steroid producing cells. In the last decade, insects have become the focus for research on the biosynthesis of the principal steroid hormones, ecdysteroids. However, the transcriptional regulatory mechanisms controlling the ecdysteroid biosynthesis genes are largely unknown. Here we show that a novel zinc finger transcription factor Ouija board (Ouib) is essential for activating the expression of one ecdysteroid biosynthesis gene, *spookier*, in the ecdysteroid producing cells. Ouib is the first invertebrate transcription factor that is predominantly expressed in the steroidogenic organs and essential for development via inducing expression of the steroidogenic gene. In addition, this is the first report showing the catalytic step-specific control of steroid hormone biosynthesis through transcriptional regulation.

## Introduction

Steroid hormones are responsible for the coordination and regulation of many biological events during development of multicellular organisms. In all species, steroid hormones are synthesized from cholesterol and/or other phytosterols by multiple steroidogenic enzymes, epitomized by the members of the steroidogenic cytochrome P450 monooxygenases. High-level steroid hormone biosynthesis generally occurs in specialized steroidogenic tissues. Thus, an important condition for achieving tissue-specificity of steroid biosynthesis is providing a regulatory mechanism that ensures tissue-specific expression of the steroidogenic enzyme genes.

In vertebrates, major sites of steroid hormone biosynthesis are the adrenal cortex, gonads and placenta, that express steroidogenic enzyme genes such as *Cyp11a1*, *P450c17a*, *3β-HSD* and *17β-HSD* [1]. Key transcriptional regulators for these genes are the orphan nuclear receptors NR5A1 and NR5A2, also known as Ad4BP/Steroidogenic Factor 1 (SF-1) and Liver receptor homolog-1 (LRH-1), respectively [2–5]. Ad4BP/SF-1 and LRH-1 are predominantly expressed in the steroidogenic cells. A collective body of previous studies has established that Ad4BP/SF-1 controls steroid hormone biosynthesis through the transcriptional regulation of all steroidogenic genes [3,5]. Moreover, forced expression of this gene is sufficient to differentiate embryonic stem cells and human induced pluripotent stem cells into the steroidogenic cells [6,7] and to induce ectopic adrenal formation [8], indicating that Ad4BP/SF-1 acts as a master regulator for steroid hormone biosynthesis in vertebrates.

In insects, the principal steroid hormones are ecdysteroids, including ecdysone and its active derivative 20-hydroxyecdysone (20E), which plays pivotal roles in controlling a number of developmental and physiological events, especially in guiding transition from one developmental stage to the next via molting and metamorphosis [9–13]. During larval and pupal development, ecdysone is synthesized from dietary cholesterol in a specialized endocrine organ called the prothoracic gland (PG). After release from the PG, ecdysone is converted to 20E in the peripheral tissues through the action of Shade, the terminal P450 monoxygenase in the biosynthetic pathway [14]. In the last 15 years, a number of genes encoding essential ecdysteroidogenic enzymes acting in the PG have been identified and characterized, including *noppera-bo* [15–17], *neverland* (*nvd*) [18,19], *Cyp307a1/spook* (*spo*) [20,21], *Cyp307a2/spookier* (*spok*) [21], *non-molting glossy/shroud* (*sro*) [22], *Cyp306a1/phantom* (*phm*) [23,24], *Cyp302a1/disembodied* (*dib*) [25,26] and *Cyp315a1/shadow* (*sad*) [26]. All of these enzymes (except *nvd* and *spok*) are collectively referred to as the Halloween genes [13,27].

Previous studies have identified multiple transcription factors essential for ecdysteroidogenic functions in the PG. For example, the Ecdysone receptor-Ultraspiracle complex and several other ecdysteroid-regulated transcription factors such as βFTZ-F1, Broad, E75A and DHR4 are involved in both forward and feedback regulation of cyclic ecdysteroid production [28–35]. Ecdysteroid biosynthesis is also transcriptionally regulated by other factors including Without children [36], Molting defective (Mld) [37], the CncC-dKeap1 complex [38], Ventral veins lacking (Vvl) [39,40], Knirps [39] and FOXO [35]. Importantly, it has been reported that Broad, CncC, dKeap1, Vvl and Knirps directly bind to enhancer regions of some ecdysteroidogenic enzyme genes [31,33,38,39].

However, it should be noted that, unlike vertebrate Ad4BP/SF-1 and LHR-1, all of the identified steroidogenic transcription factors in insects are highly expressed not only in the PG, but also in many other non-ecdysteroidogenic tissues. Furthermore, some of these transcription factors have important functions other than ecdysteroid biosynthesis. For example, FOXO is well characterized as the primary transcriptional mediator of the insulin/insulin-like peptide signaling pathway in almost all cells [41]. Similarly, the CncC-dKeap1 complex is known to regulate xenobiotic responses [42] while Vvl and Knirps play key roles in cellular differentiation and morphogenesis of several tissues during embryogenesis including the PG (i.e. [43,44]). More notably, βFTZ-F1, the insect homolog of vertebrate Ad4BP/SF-1, plays a crucial role in ecdysteroid-dependent transcriptional cascades in not only the PG but also many other tissues [4,9].

In contrast to the broad roles that all these steroidogenic factors play in other tissues during development, we describe here a much more specific role for the transcription factor coded by the gene *ouija board* (*ouib*). Ouib is a C_2_H_2_-type zinc finger transcription factor, that is specifically expressed in the *Drosophila* PG and our genetic analysis clearly demonstrates that *ouib* is only essential for the expression of Spookier (Spok), a potential rate-limiting enzyme in the ecdysone biosynthetic pathway. Most remarkable, however, is that *spok* appears to be the essential target of *ouib* since resupply of a Spok paralog in PG tissue rescues *ouib* mutants to viability. Since orthologs of *ouib* and *spok* are found only in Drosophiladae genomes, this study also suggests a presence of insect clade-specific transcriptional regulatory mechanisms of ecdysone biosynthesis.

## Results

### *CG11762/ouija board* is predominantly expressed in the prothoracic gland during embryonic and larval development

**Fig 1:**
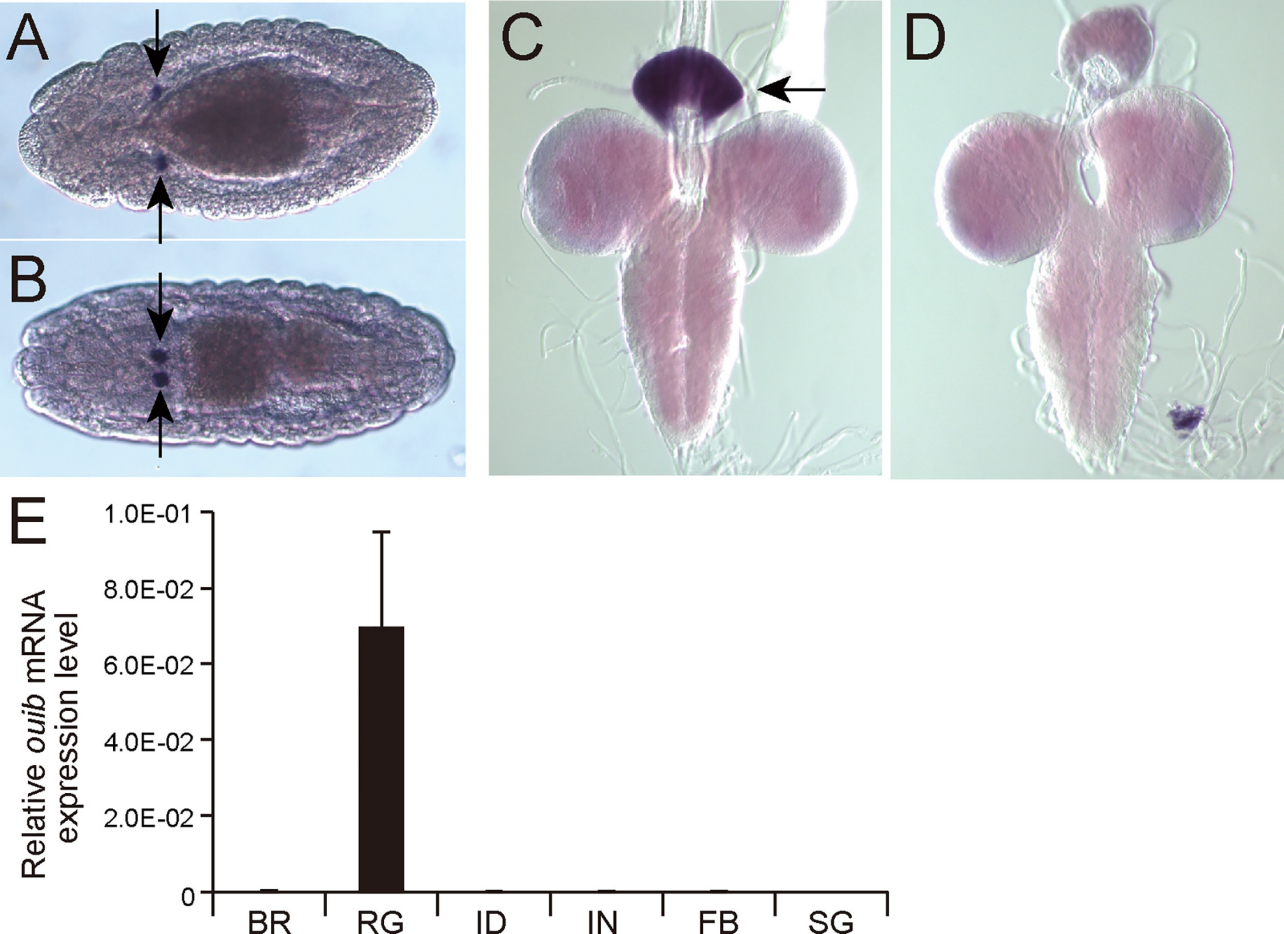
Expression analysis of *ouib* in *Drosophila* larva and embryo. (A, B) RNA *in situ* hybridization of stage 14 (A) and stage 16 (B) embryos with the *ouib* antisense RNA probe. Dorsal views are shown. *ouib* signal was detected in the primordia of PG cells (arrows). An image with sense RNA probe is shown in S1 Fig (C, D) *in situ* hybridization of third instar larval brain-ring gland complexes with the *ouib* antisense (C) and sense (D) RNA probes. *ouib* signal is detected in the ring gland including the PG cells (arrow). (E) The expression levels of *ouib* in several tissues quantified by qRT-PCR (N = 3). Total RNA was prepared from wandering third instar larvae. BR, brain; RG, ring gland; ID, imaginal disc; IN, intestine; FB, fat body; SG, salivary gland. Error bars indicate the s.e.m.

We identified *CG11762*, designated *ouija board* (*ouib*), as a gene predominantly expressed in the PG primordia in the embryonic *in situ* gene expression pattern database of the Berkeley Drosophila Genome Project Experiment ID RT01107 [45]. We confirmed the PG restricted expression of *ouib* in embryos using RNA *in situ* hybridization (Figs 1A, 1B and S1). Additional RNA *in situ* hybridization and quantitative reverse-transcription (qRT)-PCR experiments revealed that *ouib* is also predominantly expressed in the ring gland including the PG cells during larval development (Fig 1C, 1D and 1E). These results suggest that *ouib* may be involved in ecdysteroid biosynthesis.

### *ouija board* encodes a ZAD-C_2_H_2_ zinc-finger protein and is conserved only in Drosophilidae species

The predicted open reading frame of *ouib* encodes a protein that belongs to the family of the zinc-finger associated domain (ZAD) containing C_2_H_2_ zinc-finger proteins (ZFPs) [46,47]. The ZAD-ZFP family constitutes the largest subgroup of C_2_H_2_ ZFPs especially in insect species, and are characterized by an N-terminal ZAD consisting of ∼75 amino acid residues that are thought to serve as a protein-protein interaction domain [48]. In *D*. *melanogaster*, there are 98 independent loci encoding ZAD-ZFPs [47]. At least some of ZAD-ZFPs are thought to act as transcription factors, since several of them have been reported to bind DNA [49,50]. Notably, 5 paralogs of *ouib* are duplicated at the 85A9 cytological position of the third chromosome in *D*. *melanogaster* genome (S2 Fig), and one of the paralogs designated *M1BP* codes for a general transcription factor [51], raising the possibility that Ouib acts as a transcription factor in the PG.

Orthologs of *ouib* are found in genomes of 11 other Drosphilidae species (S1 Table) [52]. FlyBase (http://flybase.org/reports/FBgn0037618.html) also indicates the presence of potential orthologs of *ouib* in the mosquito species *Aedes aegypti*, *Anopheles gambiae* and *Culex quinquefasciatus*. However, a reciprocal BLAST search does not support the idea that the mosquito genomes have true *ouib* orthologs. In addition, a standard BLAST search did not detect any orthologous counterparts of *ouib* in any organisms other than Drosophilidae species. This is consistent with the previous report [47], that no orthologs of *ouib* are found in genomes of the silkworm *Bombyx mori* and the beetle *Tribolium castatenum*. Taken together, these results suggest that *ouib* is a Drosophilidae-specific ecdysteroidogenic component.

### *ouija board* is essential for larval development

**Fig 2:**
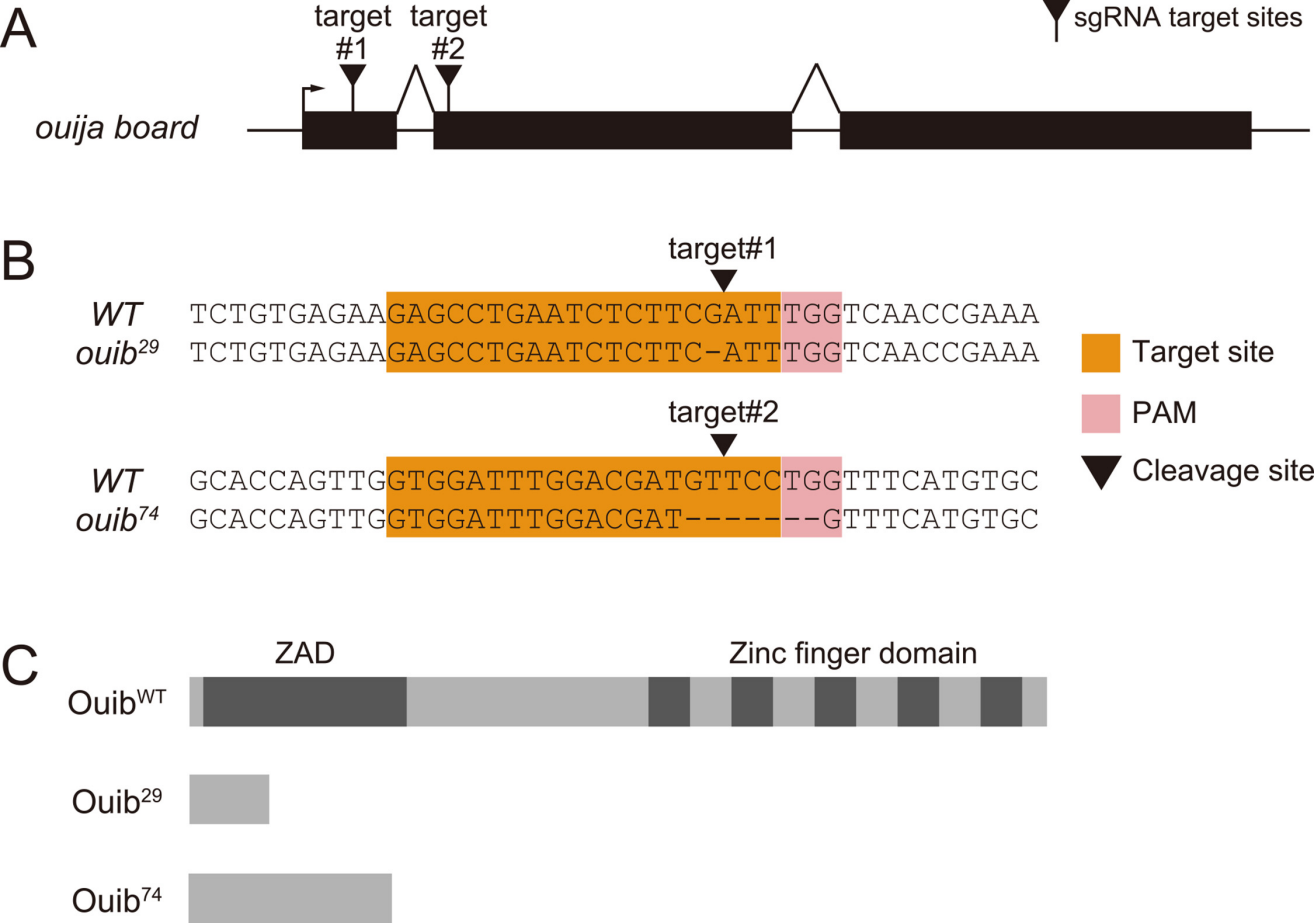
Generation of *ouib* mutant alleles by CRISPR/Cas9 system. (A) A schema of the *ouib* gene showing the sgRNA target sites. Exons are shown as black boxes, the transcriptional start site as arrow, and sgRNA target sites as black triangles. (B) Sequences of sgRNA target sites. The 20 bp target sequence corresponding to each target site is indicated in orange, along with the neighboring NGG protospacer adjacent motif (PAM) in pink and the cleavage site of Cas9 is shown as black triangles. (C) Predicted protein structures of *ouib* alleles. Ouib^29^ and Ouib^74^ are composed of 29 and 74 amino acids, respectively.

To assess the *in vivo* functional importance of *ouib*, we generated *ouib* loss-of-function alleles by a CRISPR/Cas9-dependent genome editing technology [53]. We succeeded in isolating two independent mutant alleles, *ouib* ^*29*^ and *ouib* ^*74*^, each of which had a small deletion induced by different CRISPR single guide RNAs (sgRNAs; Fig 2A and 2B). Both *ouib* ^*29*^ and *ouib* ^*74*^ alleles led to premature stop codons in the putative coding sequence of *ouib*, eliminating all 5 zinc-finger domains in the C-terminal region of Ouib (Fig 2C).

**Fig 3:**
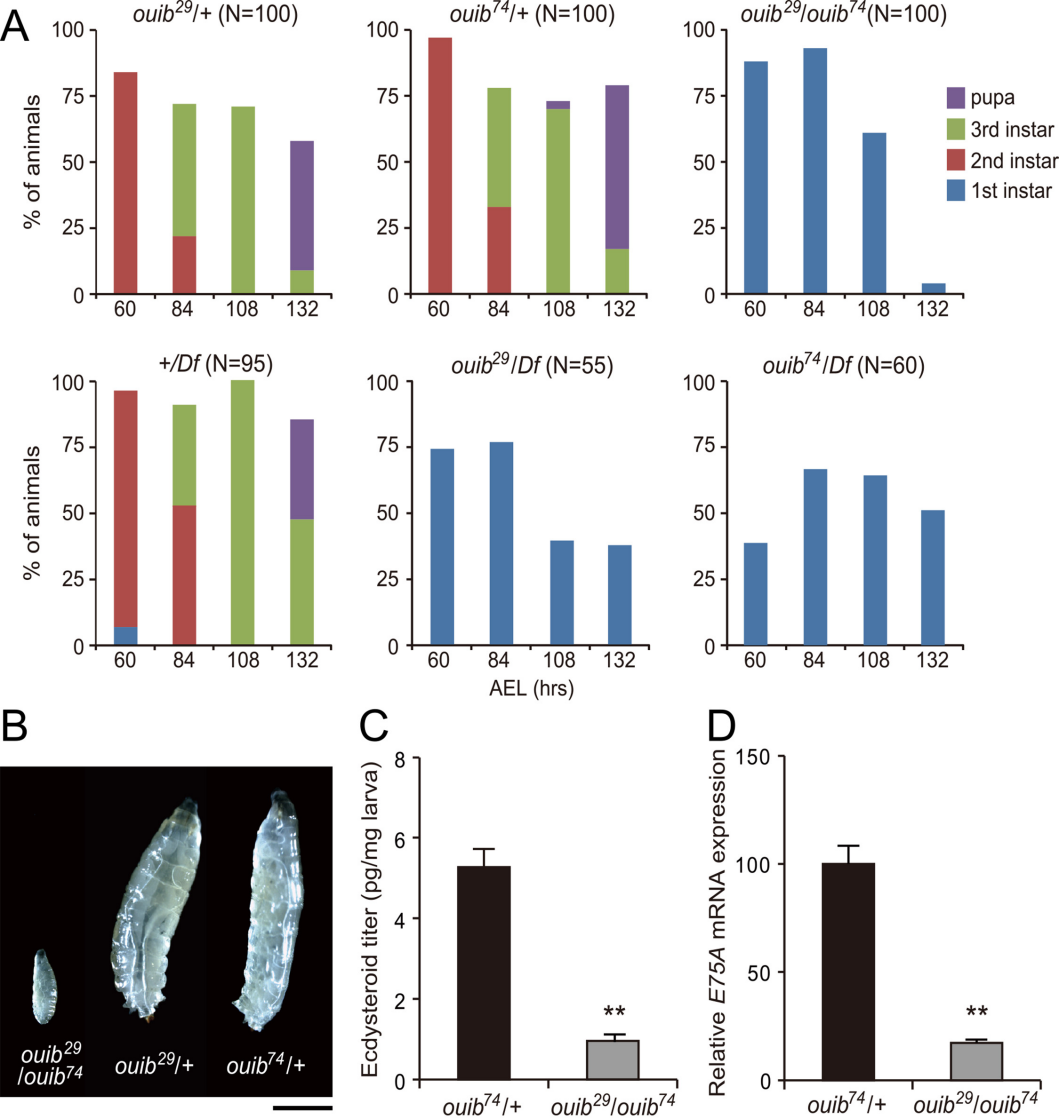
Larval lethality and developmental arrest phenotype of *ouib* mutant larvae. (A) The survival rate and developmental progression of control and *ouib* mutant animals (N = 50~100). (B) Comparison of body size and developmental stage between control (right and middle) and *ouib* mutant (left) at 108 hours AEL. Control animals became third instar larvae, while *ouib* mutant animals were first instar larvae. Scale bar: 1 mm. (C) Ecdysteroid levels in control and *ouib* mutant first instar larvae at 12 hours AH measured by ELISA (N = 4). (D) Ecdysteroid levels, as measured by the ecdysone inducible gene *E75A*, in control and *ouib* mutant first instar larvae at 36 hours AEL measured by qRT-PCR (N = 3). Error bars indicate s.e.m. **; *P*<0.01 with Student’s *t*-test.

Embryos transheterozygous for *ouib* ^*29*^/*ouib* ^*74*^ completed embryogenesis, hatched normally, and showed no apparent morphological defects after hatching. However, *ouib* ^*29*^/*ouib* ^*74*^ transheterozygotes arrested development in the first instar larval stage and, even 108 hours after egg laying (AEL) or later, never molted into second instars and (Fig 3A and 3B). Eventually all *ouib* ^*29*^/*ouib* ^*74*^ transheterozygous animals died by 144 hours AEL retaining the first instar larva-type morphology. In contrast, the majority of control *ouib* ^*29*^/+ or *ouib* ^*74*^/+ heterozygous animals became pupae (Fig 3A and 3B) by this time. To rule out the possibility that the observed phenotype was due to off-target mutations by CRISPR/Cas9 system, we combined *ouib* ^*29*^ or *ouib* ^*74*^ allele with a deficiency (Df) line that deletes a genomic region containing *ouib* locus. Similar to *ouib* ^*29*^/*ouib* ^*74*^ transheterozygotes, *ouib* ^*29*^ */Df* or *ouib* ^*74*^ */Df* animals died in the first instar stage, while *+/Df* animals were fully viable. This result provides evidence that *ouib* locus is responsible for the lethal phenotype. These results demonstrate that Ouib is essential for larval development.

### The *ouija board* loss-of-function phenotype is due to ecdysteroid deficiency

We next examined whether the larval arrest and lethality phenotype of *ouib* mutant animals was due to the loss of ecdysteroids. An ELISA assay revealed that the ecdysteroid titer in *ouib* ^*29*^/*ouib* ^*74*^ transheterozygotes was significantly reduced compared to control animals (Fig 3C). Consistent with this observation, the expression of *E75A*, which is an early ecdysteroid-inducible gene, was greatly reduced in *ouib* ^*29*^/*ouib* ^*74*^ transheterozygotes (Fig 3D). Moreover, when *ouib* ^*29*^/*ouib* ^*74*^ animals or *ouib* ^*74*^ */Df* animals were fed yeast paste containing 20E after hatching, they molted to the second instar larval stage or later, as judged by the anterior spiracular morphologies (Table 1). These results suggest that loss of *ouib* mutant phenotype is due to ecdysteroid deficiency and that *ouib* regulates ecdysteroid production in the PG during normal development.

**Table 1:** Rescue of *ouib* null mutant animals by oral administration of sterols and ecdysteroids.

Ecdy-steroid	Number of dead animals at each stage				Total number of animals	
	1st instar		2nd instar and later			
	*ouib* ^*29*^/*ouib* ^*74*^	*ouib* ^*74*^/*Df*	*ouib* ^*29*^/*ouib* ^*74*^	*ouib* ^*74*^/*Df*	*ouib* ^*29*^/*ouib* ^*74*^	*ouib* ^*74*^/*Df*
None	47	36	0	0	47	36
C	48	-	0	-	48	-
7dC	46	-	0	-	46	-
5βkd	1	-	43	-	44	-
20E	0	39	47	24	47	63

The first instar larvae of *ouib* ^*29*^/*ouib* ^*74*^ or *ouib* ^*74*^ */Df* animals were collected 36 hours AEL, and then fed yeast pastes containing 0.5% (w/w) each steroid. The number of dead animals at each stage was counted. C, cholesterol; 7dC, 7-dehydrocholesterol; 5βkd, 5β-ketodiol; 20E, 20-hydroxyecdysone; -, not determined.

### Loss of *ouija board* strongly reduces the expression of one ecdysone biosynthetic enzyme gene *spookier*

**Fig 4:**
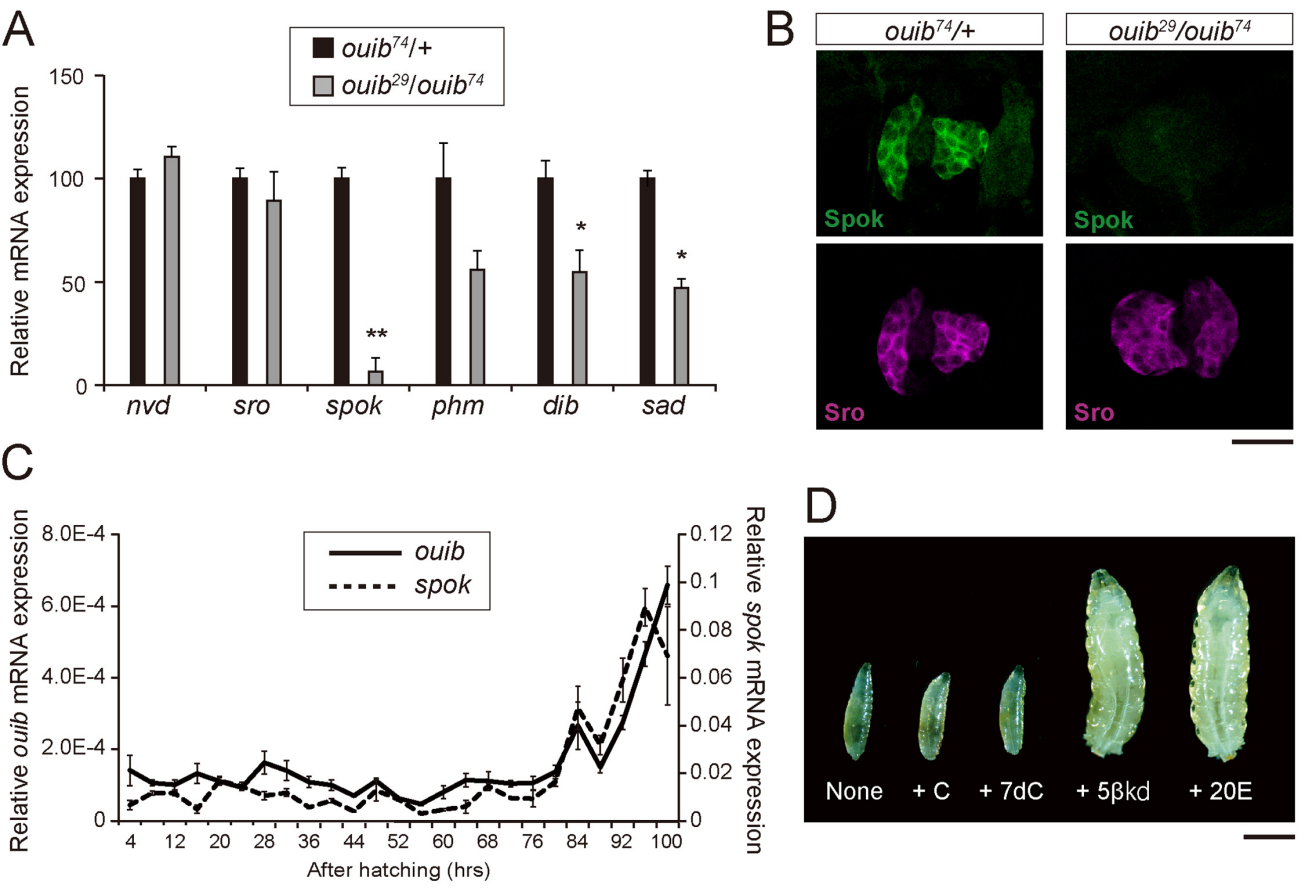
Expression analysis of ecdysteroidogenic genes and feeding rescue experiment in *ouib* mutant larvae. (A) The expression levels of ecdysteroidogenic genes in control and *ouib* mutant first instar larvae at 38 hours AEL measured by qRT-PCR (N = 3). (B) Immunostaining of the PG cells from control and *ouib* mutant first instar larvae at 36 hours AEL with antibodies against Spok (green) and Sro (magenta). Scale bar: 25 μm. (C) The transcriptional expression profiles of *ouib* and *spok* in *w* ^*1118*^ during larval development measured by qRT-PCR (N = 3). The solid line and dashed lines indicate the expression levels of *ouib* and *spok*, respectively. (D) Feeding rescue experiments for *ouib* mutant (*ouib* ^*29*^ */ouib* ^*74*^) larvae. Mutant animals fed 5β-ketodiol (5βkd) and 20-hydroxyecdysone (20E) became third instar larvae, while animals fed cholesterol (C), 7-dehydrocholesterol (7dC) and none remained first instar larvae. The lethal stages in each experimental condition were scored and shown in Table 2. Scale bar: 1 mm. Error bars indicate s.e.m. *; *P*<0.05, **; *P*<0.01 with Student’s *t*-test.

As described above, we expect that Ouib acts as a transcription factor. Considering the spatial expression pattern and the loss-of-function phenotype of *ouib*, we wondered whether loss of *ouib* resulted in changes in the expression levels of any ecdysteroidogenic genes in the PG. To address this issue, we conducted qRT-PCR experiment to examine expression levels of 6 ecdysteroidogenic genes in the first instar larvae of control and *ouib* ^*29*^/*ouib* ^*74*^ transheterozygotes. Among the 6 genes, the expression of one gene *Cyp307a2/spok* was drastically reduced in *ouib* ^*29*^/*ouib* ^*74*^ transheterozygotes as compared to control animals (Fig 4A). An immunohistological analysis using anti-Spok antibody also revealed a strong decrease of Spok protein level in *ouib* ^*29*^/*ouib* ^*74*^ larvae compared to control animals, but not that of the Sro protein, another ecdysone biosynthetic enzyme expressed in the PG. (Fig 4B). We also found that expression of *Cyp302a1/dib* and *Cyp315a1/sad*, two other ecdysone biosynthetic P450 genes, were also lower than in *ouib* ^*29*^/*ouib* ^*74*^ animals compared to control animals, but their reduction was just on the threshold of significance (Fig 4A). On the basis of the observation that the mutants cannot induce the expression of “*spookier*,” we named *CG11762* “*ouija board*” since this is an instrument for calling ghosts in western countries.

We also examined whether there was a correlation between expression of *ouib* and *spok* during larval development. Overall the expression of both genes was relatively low during early stages and gradually became higher in late stages. We also found that the temporal expression profile of *ouib* closely correlates to that of *spok* in the late third instar stage (Fig 4C). Curiously, the temporal expression profile did not always correlates to the dynamics of ecdysteroid titer during the third instar stage (S3 Fig). For example, *ouib* expression did not increase prior to white prepupal stage, when the level of ecdysteroid titer was high. This result suggests that the *ouib*-*spok* coordinated transcriptional relationship does not fully account for the temporal dynamics of ecdysteroid biosynthesis during development.

### *ouija board* plays an essential role in the “Black Box” where *spookier* has a crucial function

A previous study reported that Spok plays a crucial role in the “Black Box”, which consists of the conversion steps from 7-dehydrocholesterol (7dC) to 5β-ketodiol (5βkd) in the ecdysteroid biosynthetic pathway [21]. The participation of Spok in the “Black box” reactions was inferred by the observation that the larval arrest phenotype of *spok* RNAi animals was rescued by oral administration of 5βkd, but not 7dC or the most upstream precursor cholesterol [21]. Indeed, the same tendency as observed in *spok* RNAi animals was found in *ouib* loss-of-function animals. When *ouib* ^*29*^/*ouib* ^*74*^ transheterozygotes were fed yeast paste supplemented with cholesterol or 7dC, the larvae still arrested at the first instar larval stage (Fig 4D and Table 1). In contrast, we found that the first instar larval arrest phenotype of *ouib* ^*29*^/*ouib* ^*74*^ transheterozygotes was rescued when the animals were fed yeast paste supplemented with 5βkd (Fig 4D and Table 1). These results suggest that loss of *ouib* function specifically impairs the catalytic conversion that takes place during the “Black Box” reactions. These results also imply that the moderate reduction seen in *dib* and *sad* expression does not contribute in a major way to the *ouib* mutant phenotype.

### The *ouija board* mutant phenotype is due to loss of *spookier* expression in the prothoracic gland

In addition to the feeding rescue experiment, we examined whether the *ouib* mutant phenotype was rescued by forced expression of *spok* using GAL4-UAS binary gene expression system. We first established *UAS-spok* transgenic strains to drive *spok* expression in the PG cells under control of *phm-GAL4#22* driver. However, for an unknown reason, none of our *UAS-spok* transgenes was expressed in the PG of first and second instar larvae with the *phm-GAL4#22* driver and thus these constructs were not suitable for our experimental purpose. Therefore, we decided to examine whether the *ouib* mutant phenotype could be rescued by forced expression of *Cyp307a1/spo*, a paralog of *spok* that appears to provide the same enzymatic activity but only in embryos and in the follicular cells of the ovary [20,21]. We confirmed that *spo* was functionally equivalent to *spok in vivo*, as *spo* overexpression rescued the first instar larval arrest phenotype of *spok* RNAi animals (S2 Table). Indeed, *spo* overexpression in the PG rescued the larval arrest phenotype of *ouib* ^*29*^/*ouib* ^*74*^ transheterozygotes, and some of the animals grew up to the adult stage (Table 2). These results strongly suggest that the developmental arrest phenotype of *ouib* mutant is due solely to loss of *spok* expression in the PG. Our data therefore support the idea that Ouib is a special transcription factor primarily required for inducing expression of one biosynthetic gene *spok*, and no other essential gene during development.

**Table 2:** Rescue of *ouib* mutant animals by *spo* overexpression in the PG.

Transgenes		Number of *ouib* ^*29*^ */ouib* ^*74*^ adults		
	*Tb*	3rd instar larvae	Pupae	Adults
*phm-GAL4#22*, *UAS-spo*	+	169 (100%)	124 (73.4%)	29 (17.2%)
	-	246	n.d.	n.d.
*phm-GAL4#22*	+	0	0	0
	-	85	n.d.	n.d.
*UAS-spo*	+	0	0	0
	-	84	n.d.	n.d.

The number of *ouib* ^*29*^ */ouib* ^*74*^ animals that grew up to the third instar larval, pupal and adult stages was scored. *Tb+* indicates *ouib* ^*29*^ */ouib* ^*74*^ animals, while *Tb-* indicates *ouib* ^*29*^ */TM6* or *ouib* ^*74*^ */TM6* animals from the parental strains in the same experimental batches. *Tb-* animals can be used as internal controls. Values in parentheses indicate the percentage of animals that survive to the pupal and adult stages. The animals were fed standard cornmeal food without any steroidal supplements. Detailed genetic crosses for this experiment are described in Materials and Methods. n.d., not determined.

### Identification of Ouija board-response element in the *spookier* enhancer region

**Fig 5:**
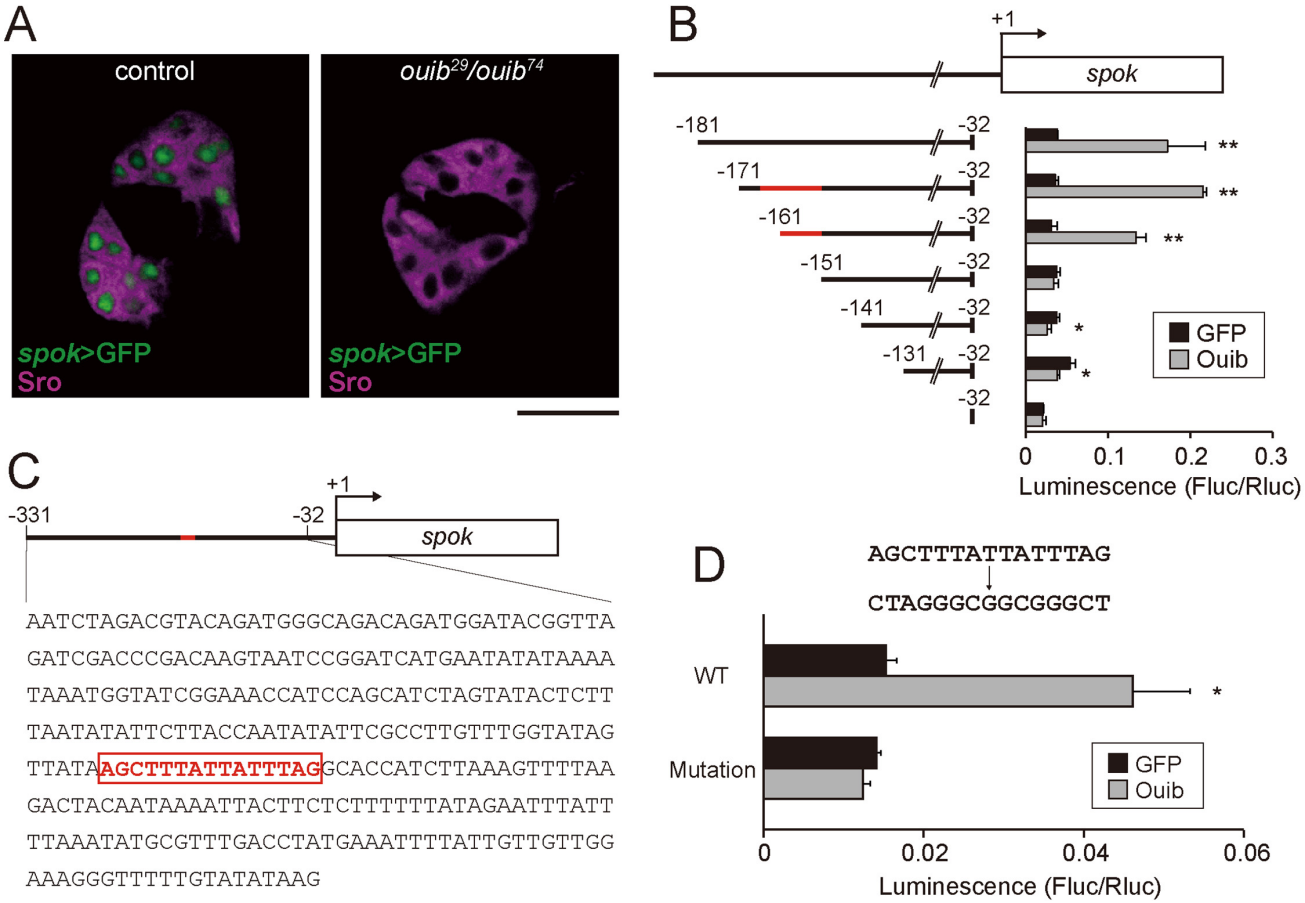
Transcriptional activity of Ouib for the upstream element of *spok*. (A) Fluorescence images of the PG cells from control and *ouib* mutant larvae with *spok* enhancer/promoter-driven nuclear localized-GFP construct (*spok>GFP*) at 36 hours AEL. PG cells are immunostained with antibody against Sro (magenta). Scale bar: 20 μm. (B) Luciferase reporter assay with plasmids containing the series of upstream elements of *spok*. Numbers indicate the distance from the translation initiation site (+1) of *spok*. The red bar indicates the Ouib-response element (-166 to -152). The white box represents the coding regions. Reporter activities of progressive deletion constructs are shown right (N = 3). The *GFP* expression plasmid was used as a negative control. (C) Schematic representation of the location of Ouib-response element (-166 to -152) in the *spok* enhancer/promoter region. The nucleotide sequence is shown below the cartoon of *spok* gene structure. The bar and box with red color indicate the 15 bp Ouib-response element. The black bar and the box indicate the enhancer/promoter and coding region, respectively. (D) Luciferase reporter assay with plasmids containing the 15 bp transversion mutation in the -166 to -152 region of the 300 bp upstream element of *spok* (N = 3). The *GFP* expression plasmid was used as a negative control. Error bars indicate s.e.m. *; *P*<0.05, **; *P*<0.01 with Student’s *t*-test.

To address whether Ouib protein acts directly on the *spok* enhancer region to induce *spok* expression, we initially searched for an Ouib-response element in the enhancer/promoter region of *spok*. We first identified a ~1.4 kb genomic region upstream of the *spok* coding sequence that was sufficient to mimic the expression of *spok* in the PG when fused to a *GFP* reporter (Figs 5A and S4). The GFP expression driven by the ~1.4 kb *spok* enhancer region was almost completely abolished in *ouib* ^*29*^/*ouib* ^*74*^ transheterozygotes (Fig 5A), suggesting that the ~1.4 kb element contains a Ouib-response element.

In order to identify the *cis*-regulatory element(s) responsible for the Ouib-mediated control of *spok* expression, we conducted a promoter/enhancer characterization analysis in a heterologous cell culture system. We generated DNA constructs carrying the upstream region of *spok* fused with a *luciferase* (*luc*) gene cassette and then transfected *Drosophila* Schneider 2 (S2) cells using these DNA constructs with or without a plasmid for overexpressing *FLAG-ouib*. We identified a 300 bp genomic region corresponding to the region from -331 bp to -32 bp upstream of the ATG start codon of *spok* that drives expression of the *luc* reporter in S2 cells in an Ouib-dependent manner (S5 Fig). The 300 bp region was also sufficient to drive expression of a GFP reporter in the PG cells (S4 Fig). To narrow down the element(s) responsible for the Ouib-dependent expression of *spok*, we tested several constructs carrying the upstream region of *spok* with a range of deletions within the 300 bp region (S5 Fig). We first generated the deletion constructs in 50 bp increments from 5´ terminus of the 300 bp region and found that the region from -181 to -131 bp was crucial for the Ouib-dependent *luc* reporter activity (S5 Fig). We then generated the deletion constructs in 10 bp increments from 5´ terminus of the -181 to -32 region. The construct carrying the -151 to -32 region did not show any induction in *luc* reporter activity even in the presence of Ouib (Fig 5B). The construct carrying the longer 10 bp 5´ extension (-161 to -32) still retained statistical significant Ouib-dependent *luc* reporter activity. However, the fold induction of *luc* reporter activity with the -161 to -32 region was slightly reduced as compared to the -171 to -32 region or longer (Fig 5B). From these results, we hypothesized that the Ouib-response element lay between -166 to -152 bps (Fig 5C). To clarify the importance of this 15 bp region for Ouib-dependent control of gene expression, we introduced transversion mutations of the entire 15 bp sequence. This mutated construct exhibited no *luc* reporter induction in the presence of Ouib upon transfection into S2 cells (Fig 5D). We also conducted subsequent reporter assays using constructs carrying various mutations in the 15 bp sequence. None of the constructs carrying any of several 3 bp substitutions within the 15bp sequence eliminated the responsive to Ouib (S6 Fig). Therefore, we conclude that Ouib binding tolerates degeneracy throughout the 15 bp sequence (5´-AGCTTTATTATTTAG-3´).

We also examined the evolutionary conservation of the Ouib-response elements in putative *spok* enhancer regions in 12 Drosophilide species whose genome sequences have been determined [52]. EMBOSS Matcher, an algorithm to identify local similarities between two sequences [54], found sequence motifs similar to the *D*. *melanogster* Ouib-response element in almost all of the Drosophilidae species (S7 Fig). In particular, the *D*. *yakuba* putative *spok* enhancer contains exactly the same 15 bp sequence motif. In addition, in the species belonging to the subgenus Sophophora, which includes *D*. *melanogaster*, the Ouib-response element-like motifs are found in proximity (within 500 bp) to the *spok* coding region (S7 Fig). These data suggest that Ouib-like response elements are also evolutionarily conserved to some degree.

### Ouija board physically associates with the Ouija board-response element

**Fig 6:**
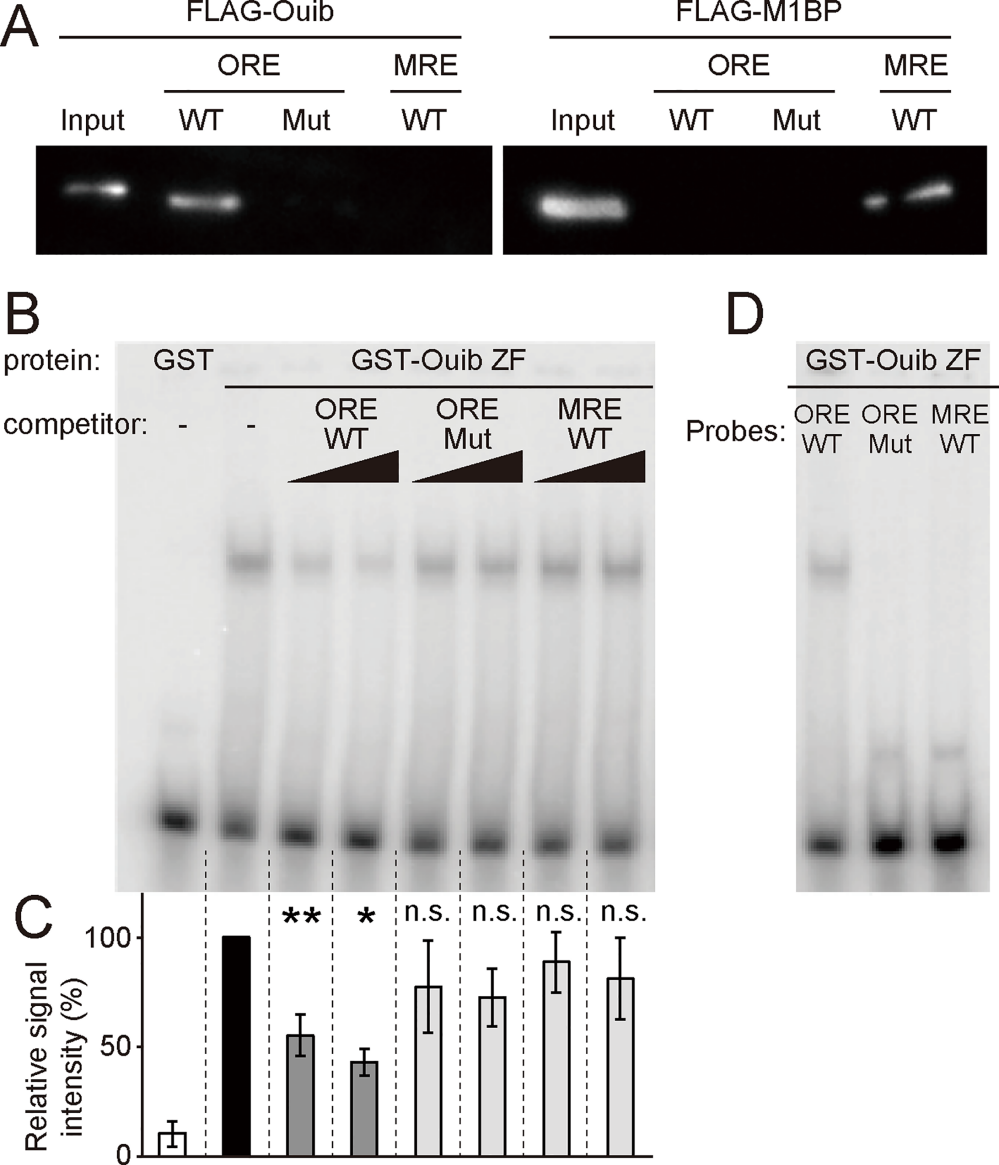
DNA-Binding analysis of Ouib for the upstream element of *spok*. (A) ABCD assay with nuclear extracts from S2 cells and avidin-biotin-conjugated double-stranded DNA probes. After pull-down, proteins were detected by western blotting using anti-FLAG antibody. (B) EMSA using recombinant proteins of GST alone or GST-fusion zinc finger domains of Ouib (amino acids 150–313) with ^32^P-labeled double-stranded oligonucleotide probes containing the wild type 15 bp Ouib-response element (ORE). The x100 and x200 amounts of the non-labeled probes of the wild type ORE (ORE WT), the mutated ORE (ORE Mut) and the wild type M1BP-response element (MRE WT) were used as cold competitors. (C) Densitometric analysis of the EMSA band radioactive intensities in the same experimental condition as B with 3 independent replicates. Average radioactivity of the ORE/GST-Ouib-Zf complex is set as 100%. Note that the complexes with the ORE were outcompeted by the unlabeled ORE WT probe, but not by the unlabeled MRE WT probe. *P<0.05 and **P<0.01 by Tukey's multiple comparisons test. n.s., not significant. (D) EMSA using recombinant proteins of GST-fusion zinc finger domains of Ouib (amino acids 150–313) with ^32^P-labeled double-stranded oligonucleotide probes of ORE WT, ORE Mut and MRE WT.

We sought to further establish if Ouib binds directly to the Ouib-response element by performing a DNA/protein binding assay. We first examined the physical interaction between the Ouib-response element sequence and Ouib protein by an ABCD assay, which uses biotin conjugated, double-stranded oligonucleotides containing the Ouib-response element sequences. Nuclear extracts obtained from S2 cells expressing *FLAG-ouib* were mixed with the biotin-labeled oligonucleotide, and then the protein-oligonucleotide complexes were pulled down using streptavidin beads. We found that FLAG-Ouib protein bound strongly to the wild type Ouib-response element probe, but not to the mutated probe (Fig 6A). In the control experiments, a biotinylated probe corresponding to M1BP (another ZAD-ZFP homolog of Ouib) binding sequence in the enhancer of *smoothened* locus [51] did not efficiently precipitate FLAG-Ouib. Conversely, FLAG-M1BP protein did not bind to the Ouib-response element, while it bound to M1BP binding element (Fig 6A).

To exclude the possibility that FLAG-Ouib protein isolated from cultured cells, indirectly associated with the probe through a complex containing some other endogenous transcription factor unrelated to Ouib, we prepared an *E*. *coli* produced recombinant protein containing the C-terminal 5 zinc finger domains (Ouib-Zf), and performed electrophoretic mobility shift assays (EMSAs) between the recombinant protein and the 15 bp Ouib-response element. We utilized 45 bp radiolabeled DNA probes, whose sequences corresponded to the *spok* enhancer region containing the 15 bp Ouib-response element. We found that the wild type oligonucleotide probes formed DNA/protein complexes with GST-Ouib-Zf, but not with GST alone (Fig 6B and 6C). In contrast, such DNA/protein complexes were not detected when radiolabeled mutated Ouib-response element sequences or sequences corresponding to the M1BP site [51] (Fig 6D) were used as probes, thereby confirming the specificity of the binding. Moreover, the complexes with the wild type 45 bp probes were outcompeted by unlabeled 45 bp DNA probes with the wild type Ouib-response element sequences, but not by the unlabeled mutated DNA probes or by the unlabeled M1BP probes (Fig 6B and 6C). Taken together, these findings strongly support the idea that Ouib specifically regulates *spok* transcription by direct binding to the Ouib-response element in the *spok* enhancer.

### *ouija board* transcript level is not affected by PTTH signaling

Previous studies found that the increase of *spok* expression in the late third instar larvae is positively controlled by prothoracicotropic hormone (PTTH) [55,56]. We therefore examined whether *ouib* expression changed in response to down regulation of PTTH signaling. However, when the levels of the PTTH receptor gene *torso* were knocked down in the PG by RNAi, we observed no change in *ouib* expression, suggesting that PTTH regulation of *spok* is not mediated through *ouib* (S8 Fig).

## Discussion

In this study, we have demonstrated that the ZAD-ZFP Ouib is required for ecdysteroid biosynthesis in the PG during *D*. *melanogaster* development. The following points summarize our finding. First, *ouib* is predominantly expressed in the PG during embryonic and larval stages. Second, *ouib* null mutants exhibit early (first instar) larval developmental arrest due to a low ecdysteroid titer. Third, the larval arrest phenotype is caused by a failure of *spok* expression in the PG, and is rescued by sole overexpression of a *spok* paralog. Finally, a specific Ouib-response element that binds Ouib was identified in the enhancer region of *spok*. Our study reports on the discovery of the first invertebrate tissue-specific, steroidogenic transcription factor.

*ouib* mutants exhibit a drastic reduction of *spok* expression. However, we point out that *ouib* mutants also show a mild statistically-significant reduction of *dib* and *sad* (Fig 4A). In fact, while no DNA sequences exactly matching the *spok* Ouib-response element (5´-AGCTTTATTATTTAG-3´) are found elsewhere in *D*. *melanogaster* genome, a number of degenerate sequences do exist in the genome, including the regions upstream of ecdysteroidogenic gene coding regions (S9 Fig). Considering the fact that the luciferase constructs carrying any of several 3 bp substitutions within the 15bp sequence are still responsive to Ouib (S6 Fig), we cannot completely rule out the possibility that Ouib is also involved in direct transcriptional regulation of genes other than *spok*, particularly *dib* and *sad*. Nevertheless, our results indicate that the impairment of expression of *dib* and *sad* seems not to contribute to the *ouib* phenotype in a major way. First, the arrest during the first instar larval stage of *ouib* mutants is rescued by oral administration of 5βkd. Since Dib and Sad play roles in the terminal hydroxylation steps downstream of the conversion of 5βkd to ecdysone [23,24,26], this finding suggests that the enzymatic levels of Dib and Sad are still sufficient to make functional levels of ecdysone. Second and more importantly, the first instar larval arrest phenotype of *ouib* mutants is rescued by a sole overexpression of *spo*, which is functionally equivalent to *spok*. Therefore, in addition to the PG specificity, we would argue that the key additional feature of Ouib is its specific role in *spok* expression. To further clarify the extent to which Ouib regulates other genes, and the functional importance of these genes, will require additional studies including transcriptome analysis/ChIP-seq analysis together with eventual mutational analysis of any identified targets.

Curiously, the presence of *ouib* only in the Drosophilidae genomes is concordant with the Drosophilidae-specific duplication of *Cyp307a* P450 subfamily. While members of the *Cyp307* P450 subfamily, which includes *spok*, are found in all arthropod species examined so far [20,21,57,58], Drosophilidae *Cyp307* genes have been duplicated within the *Drosophila* radiation [21,59]. In the case of *D*. *melanogaster*, the duplicated *Cyp307* genes are *Cyp307a1/spo* and *Cyp307a2/spok*, which are sub-functionally divergent in terms of gene expression pattern; *spo* is expressed in early embryogenesis and oogenesis, while *spok* is expressed in the PG cells in late embryogenesis as well as the larval and pupal stages [20,21]. Our data demonstrate that the spatiotemporal expression pattern of *ouib* closely matches that of *spok* but not *spo*. Notably, neither *ouib* nor *spok* transcripts are detected in embryonic stages 5–9 when the embryonic ecdysteroid titer is maximal [21,45], indicating that these genes do not contribute to producing embryonic ecdysteroids. Therefore, an acquisition of *ouib* might be a critical event for the sub-functionalization of two *Cyp307* genes by changing the regulation of their expression during the Drosophilidae evolution. In terms of evolution, it is worth mentioning that there is a case where evolution changed the activity of a single ecdysteroidogenic enzyme (Nvd) dramatically limiting the food source of *Drosophila pachea* to a single species of cactus [60]. Further assessment of the biological and evolutionary roles of *ouib* and *spok* will require determining which transcription factors are involved in the transcriptional regulation of *D*. *melanogaster spo* and *Cyp307a* genes in other insects. Since there are many divergent ZAD-ZFP genes in each insect genome and they are expanded in insect lineage-specific manner [47], it is possible that a different ZAD-ZFP gene whose primary structure is not orthologous to *ouib* could be a transcription factor for other *Cyp307a* genes.

Regarding the evolutionarily aspect of *ouib*, it is important to recognize that *spok* expression is regulated by another ZAD-ZFP called Molting defective (Mld) [21,37,39]. Interestingly, just like *ouib*, *mld* genes are also found only in genomes of Drosophilidae but not other insects [21,37]. In contrast to *ouib*, Mld does not appear to be specific for the regulation of *spok* expression. First, Mld, unlike *ouib*, is expressed in several other tissues during development besides the PG [37]. Second, Mld is essential for regulating expression Nvd as well as *spok* and perhaps other genes [39]. Third and most important, the *mld* loss–of-function phenotype is not rescued by overexpressing either *spo* or *spok* [21]. Therefore, Ouib and Mld overlap in their regulation of *spok* expression, but also have distinct functions during development. While it is still unclear whether Mld is a transcription factor, it would be intriguing to examine a functional relationship between Ouib and Mld for induction of *spok* expression in the PG. According to our qRT-PCR data, it is less likely that Mld controls *ouib* expression in the PG (S10 Fig).

Another question to be answered is how *ouib* expression is regulated during larval development. As shown above, it does not seem to be by PTTH. However, recent work as shown that *spok* and other ecdysteroidogenic enzyme genes are also influenced by humoral factors such as TGFβ/Activin [61] and monoaminergic tropic factors [62,63]. It will be interesting to determine whether these factors affect *spok* expression in the PG through modulation of *ouib* levels.

An additional significant aspect of this work is to provide the first evidence for the existence of a catalytic step-specific transcriptional regulation of steroid hormone biosynthesis in organisms. Whereas the substrate of Spok and its product have not yet been identified, Spok appears to play a crucial role in the “Black Box” step of ecdysteroid biosynthetic pathway, and it is a strong candidate for acting as a rate-limiting enzyme in the pathway [10,21,64]. Interestingly, a recent study has reported that pre-mRNA splicing of *spok*, but not any other ecdysteroidogenic genes expressed in the PG, seems to specifically depend on a protein encoded by *ecdysoneless* (*ecd*), whose mutant phenotype includes ecdysteroid deficiency [65]. Thus, a rate-limiting step of ecdysteroid biosynthesis catalyzed by Spok could be under tight control by both specific transcriptional and post-transcriptional mechanisms. Currently, it is unknown whether such catalytic-specific transcriptional and/or posttranscriptional mechanisms also exist in other organisms including vertebrates.

Similar to ecdysteroids, vertebrate steroid hormones are synthesized via several intermediates by multiple steroidogenic enzymes. Among them, the rate-limiting step in vertebrate steroid hormone productions is the delivery of substrate cholesterol from the outer mitochondrial membrane to the inner one and the subsequent conversion of cholesterol to pregnenolone by CYP11A1. It is attractive to hypothesize that the rate-limiting step in vertebrate steroid hormone biosynthesis is also specifically regulated by unidentified transcriptional and/or splicing regulator(s). Whereas no apparent orthologs of *ouib* are found in vertebrates, their genomes possess a ZAD-ZFP gene called *ZFP276*, which is a tumor suppressor gene [66]. Interestingly a *ecd* ortholog is also found in humans and may also contribute to the malignancy of certain tumor types [65]. It would be worth examining roles of these genes in steroid hormone biosynthesis in vertebrates.

## Materials and Methods

### *Drosophila* strains

*Drosophila melanogaster* flies were reared on standard agar-cornmeal medium at 25°C under a 12:12 h light/dark cycle. *w* ^*1118*^, *yw* and *Oregon R* were used as the wild type strain. *phm–GAL4#22* [55] and *w; UAS-dicer2; phm-GAL4#22/TM6 Ubi-GFP* was used as the strain to drive forced gene expression in the PG. *UAS-spo* [20] and *UAS-spok-IR* [21] transgenic flies were obtained from Hiroshi Kataoka (The University of Tokyo) and Hajime Ono (Kyoto University), respectively. *y* ^*1*^ *v* ^*1*^ *nos-phiC31; attP40*, *v* ^*1*^ and *y* ^*2*^ *cho* ^*2*^ *v* ^*1*^ *; attP40{nos-Cas9}/CyO* [53] were obtained from National Institute of Genetics, Japan. The *w; sna* ^*Sco*^ */CyO; P{w+mC = tubP-GAL80* ^*ts*^ *}7* (stock number #130453) and *w* ^*1118*^ *; Df(3R)ED5330/TM6C Sb* ^*1*^, a deficiency strain that deletes a genomic region including the *ouib* locus (stock umber #150241) [67], were obtained from Drosophila Genetic Resource Center. *UAS-torso-IR* (stock number #101154) and *UAS-mld-IR* (stock number #17329) were obtained from the Vienna *Drosophila* RNAi center.

### *in situ* RNA hybridization

Digoxygenin (DIG)-labeled antisense RNA probes were synthesized using DIG RNA labeling mix (Roche) and T3 and T7 RNA polymerase (Fermentas). To generate the *ouib* probe, the *ouib* ORF was amplified by PCR with cDNA derived from whole bodies of *Oregon R* larvae and the primers described in S3 Table. PCR product was inserted into *Sma*I-digested pBluescript II SK (-), and then used as the templates for synthesizing RNA probes. Fixation, hybridization and detection were performed as [23,68].

### Quantitative reverse transcription (qRT)-PCR

RNA was isolated using the RNAiso Plus reagent (TaKaRa). Genomic DNA digestion and cDNA synthesis were performed using the ReverTra Ace qPCR RT Kit (TOYOBO). qRT-PCR was performed using the THUNDERBIRD SYBR qPCR Mix (TOYOBO) or Universal SYBR Select Master Mix (Applied Biosystems) with a Thermal Cycler Dice TP800 or TP870 system (TaKaRa). Serial dilutions of a plasmid containing the ORF of each gene were used as a standard. The expression levels of the target genes were normalized to an endogenous control *ribosomal protein 49* (*rp49*) in the same sample. The primers for quantifying *D*. *melanogaster ouib* and *E75A* are described in S3 Table. Primers amplifying *nvd*, *sro*, *spok*, *phm*, *dib*, *sad* and *rp49* were previously described [22,55].

### Immunostaining

Tissue dissections were performed in PBS followed by fixation in 4% PFA for 20 minutes at room temperature. For this study, the following primary antibodies were: mouse anti-FLAG M5 (1:1,000) (Sigma); rabbit anti-Phm (1:200) [30], guinea pig anti-Spok (1:200) [61]; guinea pig anti-Sro (1:1,000) [62]. Tissues were incubated over night with primary antibodies at 4°C. Fluorescent conjugated secondary antibodies used in this study, goat anti-mouse Alexa Fluor 488, goat anti-guinea pig Alexa Fluor 488, goat anti-rabbit Alexa Fluor 555 and goat anti-guinea pig Alexa Fluor 555, were purchased from Life Technologies. Secondary antibodies were diluted 1:500 and incubated for 1 hour at room temperature. Confocal images were captured using Carl Zeiss LSM 700 laser scanning microscope.

### UAS vectors, overexpression of genes and generation of transgenic strains

The GAL4-UAS system [69] was used to overexpress genes in *D*. *melanogaster*. To generate pUAST vector to overexpress *ouib*, specific primers including a sequence coding FLAG tag at N terminal were used for PCR to add *EcoR*I and *Xba*I sites to the 5´ and 3´ ends, respectively (S3 Table). Template cDNAs were reverse transcribed using total RNA of the ring gland from *D*. *melanogaster* using ReverTra Ace qPCR RT Kit (TOYOBO). PCR was performed using KOD Plus Neo (TOYOBO). The amplified CDS region of *ouib* was digested with *EcoR*I and *Xba*I, and then ligated into a pWALIUM10-moe vector [70]. Transformants were established by BestGene, Inc.

### Generation of the *ouib* alleles

Generation of the *ouib* allele was carried out by CRISPR/Cas9 system using the pBFv-U6.2 vector [53] provided by the National Institute of Genetics, Japan. We selected 2 independent target sites (target#1 and target#2 as shown in Fig 2). To minimize off-target effects of CRISPR/Cas9 system, we confirmed by BLAST search that no 15 nucleotide stretches within the selected target sequence (23 nucleotides including PAM motif) matched any other sequence on the 3rd chromosome. Sense and antisense oligonucleotides corresponding to sgRNA target sequences (S3 Table) were annealed and inserted into *BbsI*-digested pBFv-U6.2 vector. The *ouib* sgRNA vectors were injected into the embryos of the *y* ^*1*^ *v* ^*1*^ *nos-phiC31; attP40* strain. The *nos-Cas9*-based gene targeting was carried out as previously described [53]. Males carrying *nos-Cas9* and a sgRNA transgene were crossed to wild-type flies by mass mating. From their progeny, 10 and 50 single males for the target#1 and target#2 sites, respectively, were isolated. Each male was crossed with *w; TM3 Sb/TM6 Tb* females and then the independent isogenized strains were established. Among them, we surveyed the strains showing homozygous lethality and eventually 1 target#1 and 29 target#2 lethal strains were selected. To confirm indel mutations at *ouib* locus in each strain, we performed the T7EI assay as previously described [53]. In this assay, genome DNA from the heterozygous adults of each strain was extracted as previously described [53]. To amplify the DNA fragment including Cas9 target sites, PCR was conducted with KOD FX Neo (TOYOBO), the extracted genome DNA, and the primers listed in S3 Table [53]. The PCR products were treated with T7 endonuclease (NEB). The reacted samples were analyzed by agarose gel electrophoresis. Out of 30 total candidate strains, 1 target#1 and 8 target#2 strains were selected as candidate flies possessing indel mutations in *ouib* region. The PCR products from the 9 strains were subcloned into a *Sma*I-digested pBluescript II (Promega) and then sequenced with T3 and T7 primers. We detected small deletions in 8 out of the 9 strains. The minimal and maximal deletion sizes were 1 bp and 13 bp, respectively. We chose 1 strain for each target sites for further analyses and renamed them *ouib* ^*29*^ and *ouib* ^*74*^, both of which caused frameshift mutations for *ouib* locus (Fig 2).

### Scoring of developmental progression of *ouib* mutants

*ouib* ^*29*^ */TM3 Act-GFP* flies, *ouib* ^*74*^ */TM3 Act-GFP* flies and *w* ^*1118*^ flies were crossed each other. Eggs were laid on grape plates with yeast pastes at 25°C for 8 hours. 36 hours AEL, 100 hatched GFP negative (*ouib* ^*29*^ */+*, *ouib* ^*74*^ */+* and *ouib* ^*29*^ */ouib* ^*74*^) first instar larvae were transferred into vials with standard cornmeal food (25 animals per vial). Every 24 hours, developmental stages were scored by tracheal morphology as previously described [22].

### Feeding rescue experiments with ecdysteroids and intermediates

For the rescue experiments, 20 mg of dry yeast was mixed with 38 μl H_2_O and 2 μl ethanol or supplemented with 2 μl of the following sterols dissolved in ethanol: cholesterol (Wako; 150 mg/ml), 7-dehydrocholesterol (Sigma; 150 mg/ml), 5β-ketodiol (kindly gifted from Yoshinori Fujimoto, Tokyo Institute of Technology; 150 mg/ml) and 20-hydroxyecdysone (Sigma; 50 mg/ml). We crossed *ouib* ^*29*^ */TM3 Ser* ^*1*^ *GMR2 Act-GFP* flies with *ouib* ^*74*^ */TM3 Ser* ^*1*^ *GMR2 Act-GFP* flies. Eggs were laid on grape plates with yeast pastes at 25°C for 12 hours. At 36 hours AEL, 50 hatched GFP negative (*ouib* ^*29*^ */ouib* ^*74*^) first instar larvae were transferred to the yeast paste on grape plates and kept at 25°C. Every 24 hours, developmental stages were scored by tracheal morphology as previously described [22].

### Genetic rescue experiments with *ouib* and *spo*

For the rescue experiments of *ouib* mutant by *ouib* overexpression, *ouib* ^*29*^ *phm-GAL4#22/TM3 Act-GFP* was established by chromosomal recombination. The flies of *UAS-FLAG-ouib-1M; ouib* ^*74*^ */TM6 Ubi-GFP* were crossed with the flies of *ouib* ^*29*^ *phm-GAL4#22/TM3 Act-GFP*, the flies of *ouib* ^*74*^ */TM3 Act-GFP* were crossed with the flies of *ouib* ^*29*^ *phm-GAL4#22/TM3 Act-GFP*, and the flies of *UAS-FLAG-ouib-1M; ouib* ^*74*^ */TM6 Ubi-GFP* were crossed with the flies of *ouib* ^*29*^ */TM3 Act-GFP*. Eggs were laid on grape plates with yeast pastes at 25°C for 12 hours. At 36 hours AEL, 50 hatched GFP negative (*UAS-FLAG-ouib-1M/+; ouib* ^*29*^ *phm-GAL4#22/ouib* ^*74*^, *ouib* ^*29*^ *phm-GAL4#22/ouib* ^*74*^ and *UAS-FLAG-ouib-1M/+; ouib* ^*29*^ */ouib* ^*74*^) first instar larvae were transferred to the standard agar-cornmeal medium. Developmental stages were scored 108 hours AEL by tracheal morphology as previously described [22].

For the rescue experiments of *spok* RNAi by *spo* overexpression, *UAS-spok-IR UAS-spo* was established by chromosomal recombination on third chromosome. The flies of *UAS-spok-IR UAS-spo* strain was crossed with *w; UAS-dicer2; phm-GAL4#22/TM6 Ubi-GFP* flies. Eggs were laid on standard agar-cornmeal medium at 25°C for 24 hours. After 7 days, developmental stages of the animals on the wall were scored by presence of TM6 balancer.

For the rescue experiments of *ouib* mutant by *spo* overexpression, *Roi/CyO; ouib* ^*29*^ *phm-GAL4#22/TM6*, *Roi/CyO; ouib* ^*29*^ *UAS-spo/TM6* and *Roi/CyO; ouib* ^*74*^ *UAS-spo/TM6* were established by chromosomal recombination on third chromosome. The flies of *Roi/CyO; ouib* ^*29*^ *phm-GAL4#22/TM6* were crossed with *Roi/CyO; ouib* ^*74*^ *UAS-spo/TM6*, the flies of *Roi/CyO; ouib* ^*29*^ *phm-GAL4#22/TM6* were crossed with *Roi/CyO; ouib* ^*74*^ */TM6* and *Roi/CyO; ouib* ^*74*^ */TM6* were crossed with *Roi/CyO; ouib* ^*29*^ *UAS-spo/TM6*. Eggs were laid on standard agar-cornmeal medium at 25°C for 24 hours. After 7 days, developmental stages of the animals on the wall were scored by presence of TM6 balancer.

### Ecdysteroid measurement

*ouib* ^*29*^ */TM3 Ser* ^*1*^ *GMR2 Act-GFP* flies and *w* ^*1118*^ flies were crossed with *ouib* ^*74*^ */TM3 Ser* ^*1*^ *GMR2 Act-GFP* flies. Eggs were laid on grape plates with yeast pastes at 25°C and the hatched larvae were cleared. After 8 hours, GFP negative (*ouib* ^*74*^ */+* and *ouib* ^*29*^ */ouib* ^*74*^) first instar larvae were transferred into vials with standard cornmeal food. At 12 hours AH, whole larvae were rinsed in water and homogenized in 50 μl methanol and supernatant was collected following centrifugation at 14,000 rpm at 4°C. The remaining tissue was re-extracted in 50 μl methanol over night at 4°C. The supernatants were evaporated using a EYELA CVE-2000 (Tokyo Rikakikai) and redissolved in 50 μl EIA buffer [0.1 M PBS/0.1% BSA, 0.4 M NaCl, 1 mM EDTA and 0.01% NaN_3_]. ELISA was performed according to manufacturer’s instructions using 20-Hydroxyecdysone EIA Antiserum, 20-Hydroxyecdysone AChE Tracer and Ellman’s Reagent (Cayman Chemical) that detects 20-hydroxyecdysone with the same affinity. Standard curves were generated using 20E (Sigma). Absorbance was measured at 415 nm on a plate reader, Multiskan GO (Thermo Scientific) using the SkanIt Software 3.2 (Thermo Scientific).

### GFP reporter assay

To generate the *spok*>GFP reporter construct, a ~1.4 kb fragment immediately upstream of the *spok* transcription unit was amplified from *yw* genomic DNA using the primers 1.45spok-p_F and 1.45spok-p_R (S3 Table). This fragment was first subcloned into the pCR2.1-TOPO vector (Life Technologies) and then removed as an *Eco*RI fragment and cloned into the *Drosophila* transformation vector pH-Stinger [71]. To refine the location of the PG enhancer, seven 250–300 bp overlapping fragments that covered the entire 1.4 kb fragment were derived through PCR and each cloned into hH- Stinger. The only fragment that gave expression in the PG of transgenic animals was the ~300 bp fragment immediately upstream of the transcriptional start site. This fragment was generated using the primers 300spok-p_F and 300spok-p_R (S3 Table). Transgenic lines were generated through standard means using a *w* ^*1118*^ host background. The GFP reporter strains of *spok>GFP; ouib* ^*29*^ */TM6 Ubi-GFP* and *spok>GFP; ouib* ^*74*^ */TM6 Ubi-GFP* were established and crossed each other. Eggs were laid on grape plates with yeast pastes at 25°C for 4 hours. The first instar larvae were dissected 36 hours AEL and immunostained.

### Construction of luciferase reporter plasmids

The upstream regions of *spok* were amplified from *Oregon R* genomic DNA by specific primers to add *Sac*I and *Bgl*II sites to the 5´ and 3´ ends, respectively. PCR was performed using KOD Plus Neo (TOYOBO). The amplified upstream regions of *spok* were digested with *Sac*I and *Bgl*II, and then ligated into a pGL3-Basic vector luciferase reporter plasmid (Promega). Reporter plasmids carrying mutated regions were constructed from the pGL3-Basic plasmid containing WT upstream 300 bp region by inverse PCR. The primers for PCR are listed in S3 Table.

### Transfection and luciferase reporter assays

S2 cells were seeded in 1 ml Schneider’s Drosophila Medium (GIBCO) in a 24-well plate (greiner bio-one) 1 day before transfection. Transfection of S2 cells was performed using the Effectene Transfection Reagent (Qiagen). *GFP-*pUAST [23] and *FLAG-ouib*-pWALIUM10-moe plasmids were transfected, respectively, along with the *Actin5C-GAL4* construct (a gift from Yasushi Hiromi, National Institute of Genetics) and the luciferase reporter plasmids. The Copia Renilla Control plasmid (addgene; #38093) [72] was used as the reference. The cells were incubated for 2 days after transfection. Then they were processed by using the Dual-Luciferase Reporter Assay System (Promega) in accordance with the manufacturer’s instructions and were analyzed with Flash’n glow LB 955 (Berthold Technologies).

### Preparation of S2 cell nuclear extracts

S2 cells overexpressing *FLAG-ouib* or *FLAG-M1BP* were collected and washed with TBS. Cells were then centrifuged at 4000 g at 4°C for 5 min. The pellet was suspended and vortexed with 400 μl Buffer A [10 mM Hepes pH 7.9, 10 mM KCl, 1 mM DTT and 1 unit Complete Mini (Roche)] and 25 μl 10% NP-40. Then sample was centrifuged at 1500 g at 4°C for 5 min. The pellet was suspended and vortexed with 50 μl Buffer C [20 mM Hepes pH7.9, 400 mM NaCl, 2 mM MgSO_4_, 1mM DTT and Complete Mini (Roche)], then shaked at 4°C for 30 min. After shaking, sample was centrifuged at 14,000 rpm at 4°C for 5 min and supernatant was collected.

### Western blotting

Samples were boiled with SDS sample buffer [150 mM Tris-HCl pH 6.8, 0.6% SDS, 15% glycerol, 0.009 mg/μl Bromophenol blue, 5% 2-mercaptoethanol and 1 unit Complete Mini (Roche)] for 5 min, and loaded on 12% polyacrylamide gel followed by transfer onto PVDF membrane (GE Healthcare). Anti-FLAG M5 monoclonal antibody (1:1,000; Sigma) was used for primary antibody and ECL Peroxidase labeled anti-mouse antibody (1:10,000; GE Healthcare) was used for secondary antibody. The band was detected by ECL Ultra Lumigen TMA-6 (GE Healthcare) and Ez-Capture MG (ATTO).

### Avidin-Biotin-Conjugated DNA-Binding (ABCD) assay

Preparation of S2 cell nuclear extracts is described in the Supplemental Materials. ABCD assay was conducted essentially as previously described [73]. Biotin-labeled DNA probes were purchased from Life Technologies. The probes were incubated with Dynabeads M-280 Streptavidin (Life Technologies) at room temperature for 15 min. DNA-beads complexes were mixed with nuclear extracts and ABCD Binding Buffer [50 mM Hepes pH 7.9, 150 mM NaCl, 0.5% Triton X-100, 20 ng/μl poly(dI/dC)], and incubated at 4°C for 1 hour. After incubation, the beads were washed with ABCD Binding Buffer. The biotin-labeled oligonucleotides are listed in S3 Table.

### Preparation of recombinant proteins in *E coli*

GST proteins fused with or without 150–313 amino acid residues of Ouib (GST-Ouib-Zf) containing 5 zinc finger domains were expressed using pGEX-4T-3 vector system (GE Healthcare) in *Escherichia coli* BL-21 strain. *E*. *coli* cells were harvested and crashed with sonication. GST alone and GST-Ouib-Zf were purified from the supernatant with AKTA start equipped with GSTrap affinity column (GE Healthcare).

### Electrophoretic mobility shift assay (EMSA)

Electrophoretic mobility shift assay was conducted as previously described [74,75]. 45 bp double-stranded oligonucleotide probes containing wild type M1BP binding site, wild type and mutated (transversion) Ouib response element were prepared by annealing single-strand oligonucleotides listed in S3 Table. The wild type M1BP binding site was derived from the *smoothened* promoter [51]. Double-stranded DNA fragment was end-labeled by using T4 polynucleotide kinase (TOYOBO) and [γ-^32^P]ATP. GST or GST-Ouib fusion proteins (400 ng) were incubated for 30 min at 4°C in the reaction mixture [12 mM Hepes, pH 7.9, 1 mM dithiothreitol, 1 mM EDTA, 60 mM KCl, 4 mM MgCl2, 2 mM ZnSO4, 50 ng/ul poly(dI-dC), 1 mg/ml BSA and 12% Glycerol] in the presence or absence of 100–200-fold molar excess of specific double-stranded competitor DNA. A radiolabeled DNA probe (0.3 ng, 40,000 cpm) was added, and the incubation was continued for 20 min at 4°C. The incubation mixture was directly loaded on a 5% non-denaturing polyacrylamide gel in 1 × TBE buffer [89 mM Tris-HCl, pH 8.0, 89 mM boric acid, and 2 mM EDTA], and electrophoresed at 4°C with buffer circulation. The gels were dried and analyzed with a bio-imaging analyzer Typhoon 8600 (Amersham Pharmacia Biotech Inc). The competitor oligonucleotides used are listed in the S3 Table.

## Supporting Information

S1 Table Drosophilidae orthologs of *ouija board*. The orthologs of *ouija board* in 12 Drosophilidae species are described in the FlyBase website (http://flybase.org/reports/FBgn0209782.html). We confirmed by the BLAST search that the amino acid sequence of any of these putative proteins as a query is most similar to that of *D*. *melanogaster* CG11762.
(PDF)

S2 Table Rescue of *spok* RNAi animals by *spo* overexpression in the PG. The number of *spok* RNAi animals that grew up to the 3rd instar larval stage or later stage was scored. Detailed genetic crosses for this experiment are described in Materials and Methods. The animals were fed standard cornmeal food without any steroidal supplements. Values in parentheses indicate the number of control non-RNAi progeny from the parental strains in the same experimental batches.
(PDF)

S3 Table List of primers/oligonucleotide DNAs used in this study. Small letters indicate the restriction enzyme recognition sequences. Under lines indicate the transversion mutation sequences. Asterisks indicate 5´ biotinylation.
(PDF)

S1 Fig RNA *in situ* hybridization using embryos with antisense and sense *ouib* RNA probes. Dorsal views are shown. (A) Signals with antisense probe. (B) Signals with sense probe. Arrows indicate positions of the PG primordia. Scale bar: 100 μm.
(PDF)

S2 Fig The genomic structure of *ouija board* (*CG11762*) and surrounding genes. The data are derived from the FlyBase GBrowse website (http://flybase.org/cgi-bin/gbrowse2/dmel/?Search=1;name=FBgn0037618). Numbers indicate the nucleotide positions at the 85A9 cytological position of the chromosome 3R scaffold. Boxed arrows represent gene spans and their directions. The 5 ZAD-ZNF genes are colored by magenta.
(PDF)

S3 Fig The temporal expression profiles of *ouib* expression and ecdysteroid levels during the third instar larval development. *ouib* expression and ecdysteroid levels in *w* ^*1118*^ during the 3rd instar stage measured by qRT-PCR (N = 3) and ELISA (N = 4). The blue line indicates the relative expression level of *ouib*, normalized to the level of 0–6 hours after L2-L3 molting (0–6 hr A3L). Error bars indicate the s. e. m.
(PDF)

S4 Fig GFP expression driven by the *spok* enhancer. (A, B) Phase-contrast (left) and fluorescence (right) images of the 108 hours AEL 3rd instar larval brain-ring gland complexes with *spok*>GFP construct. The PG cells were immunostained with anti-Sro antibody (magenta). The *spok*>GFP constructs contain 1.45 kbp (A) and 300 bp (B) enhancer regiosn of *spok*, respectively. Scale bar: 100 μm.
(PDF)

S5 Fig Luciferase reporter assay with plasmids containing the series of upstream elements of *spok*. Numbers indicate the distance from the translation initiation site (+1) of *spok*, and white box represents the coding region of *spok*. *Luc* reporter activities of progressive deletion constructs are shown in right. Bars and error bars represent the average and the s. e. m., respectively, of three independent experiments. **; *P*<0.01 by Student’s *t*-test.
(PDF)

S6 Fig Luciferase reporter assay with plasmids containing the triplet transversion mutations in the -166 to -152 region of the 300 bp upstream element of *spok*. The introduced transversion mutations in the 1’, 2’, 3’, 4’ and 5’ constructs are shown in the top. The *GFP* expression plasmid was used as a negative control. Bars and error bars represent the average and the s. e. m., respectively, of three independent experiments. **; *P*<0.01 by Student’s *t*-test.
(PDF)

S7 Fig The evolutionary conservation of sequences similar to Ouib-response element in putative *spok* enhancer/promoter regions of 12 Drosophilidae species. EMBOSS Matcher [54] was used to search for sequences similar to the *D*. *melanogaster* Ouib response element (15 bp) within the 1 kb regions upstream of the translation initiation site of the *spok* loci from 12 Drosophilidae species. Numbers before and after nucleotide sequences indicate the distance from the translation initiation site of *spok*. Parentheses indicate numbers of identical matches to *D*. *melanogaster* Ouib response element. “S” and “D” indicate the subgenera Sophophora and Drosophila, respectively. *spok* genes are *DG27210* (*D*. *simulans* #1), *GD27133* (*D*. *simulans* #2), *GD28291* (*D*. *simulans* #3), *GM22791* (*D*. *sechellia*), *GG16659* (*D*. *erecta*), *GE19452* (*D*. *yakuba*), *GF20000* (*D*. *ananassae*), *GA31537* (*D*. *pseudoobscura*), *GL21970* (*D*. *persimilis*), *GK19177* (*D*. *willistoni*), *GI23968* (*D*. *mojavensis*), *GH21174* (*D*. *grimshawi*) and *GJ26360* (*D*. *virilis*). Note that a BLAST search using *D*. *melanogaster* Spok protein sequence as a query hit 3 *D*. *simulans spok* candidate genes. Also note that the BLAST search hit only one spo/spok family gene in *D*. *grimshawi* genome and thus it is not faithfully judged if *GH21174* is orthologous to *spo* or *spok*.
(PDF)

S8 Fig Expression level of *ouib* in the PGs of control and *torso* RNAi third instar larvae. Amounts of *ouib* mRNAs were measured by qRT-PCR. *phm>+* and *phm>torso-IR* indicate *w* ^*1118*^ *; +/+; phm-GAL4#22/+* and *w* ^*1118*^ *; UAS-torso-IR/+; phm-GAL4#22/+*, respectively. RNA samples were collected 140 hours after egg laying. Bars and error bars represent the average and the s. e. m., respectively, of three biological replicates. n.s. means *P*>0.05 by Student’s *t*-test.
(PDF)

S9 Fig Sequences similar to Ouib-response element in putative enhancers/promoters of ecdysteroidogenic genes in *D*. *melanogaster*. EMBOSS Matcher [54] was used to search for sequences similar to *D*. *melanogaster* Ouib response element (15 bp) within the putative enhancer/promoter regions of *D*. *melanogaster* ecdysteroidogenic enzyme genes. Numbers before and after nucleotide sequences indicate the distance from the translation initiation site of each gene. Parentheses indicate numbers of identical matches to *D*. *melanogaster* Ouib response element. Except for *phm*, a enhancer/promoter region was defined as a genomic region between the translation initiation site of each ecdysteroidogenic enzyme gene and the 3´ end of a gene next to the enzymatic gene. A *phm* enhancer/promoter is a -500 to -1 region as previously characterized [31].
(PDF)

S10 Fig Expression level of *spok* and *ouib* in the PGs of control and *mld* RNAi first instar larvae. Amounts of *spok* and *ouib* mRNAs were measured by qRT-PCR. *phm>dicer2* and *phm>dicer2+mld-IR* indicate *w* ^*1118*^ *; UAS-dicer2/+; phm-GAL4#22/+* and *w* ^*1118*^ *; UAS-dicer2/UAS-mld-IR; phm-GAL4#22/+*, respectively. RNA samples were collected 36 hours after egg laying. Bars and error bars represent the average and the s. e. m., respectively, of three biological replicates. ** and n.s. mean *P*<0.01 and *P*>0.05 by Student’s *t*-test, respectively.
(PDF)
