# Supplementary material for: The Drosophila Zinc Finger Transcription Factor Ouija Board Controls Ecdysteroid Biosynthesis through Specific Regulation of spookier
Source: PLoS Genet. 2015 Dec 10;11(12):e1005712. doi: 10.1371/journal.pgen.1005712 (PMC4684333; doi:10.1371/journal.pgen.1005712)
Supplement: S1 Table — The orthologs of ouija board in 12 Drosophilidae species are described in the FlyBase website (http://flybase.org/reports/FBgn0209782.html). We confirmed by the BLAST search that the amino acid sequence of any of these putative proteins as a query is most similar to that of D. melanogaster CG11762. (PDF) [file pgen.1005712.s001.pdf]

S1 Table.

Komura-Kawa et al.

Species names	Gene names
D. melanogaster	*CG11762*
D. simulans	*GD18569*
D. sechellia	*GM23758*
D. erecta	*GG12626*
D. yakuba	*GE25902*
D. ananassae	*GF18185*
D. pseudoobscura pseudoobscura	*GA11182*
D. persimilis	*GL12476*
D. willistoni	*GK11273*
D. virilis	*GJ22678*
D. mojavensis	*GI22393*
D. grimshawi	*GH18576*
