# Supplementary material for: The Drosophila Zinc Finger Transcription Factor Ouija Board Controls Ecdysteroid Biosynthesis through Specific Regulation of spookier
Source: PLoS Genet. 2015 Dec 10;11(12):e1005712. doi: 10.1371/journal.pgen.1005712 (PMC4684333; doi:10.1371/journal.pgen.1005712)
Supplement: S2 Table — The number of spok RNAi animals that grew up to the 3rd instar larval stage or later stage was scored. Detailed genetic crosses for this experiment are described in Materials and Methods. The animals were fed standard cornmeal food without any steroidal supplements. Values in parentheses indicate the number of control non-RNAi progeny from the parental strains in the same experimental batches. (PDF) [file pgen.1005712.s002.pdf]

S2 Table.

Komura-Kawa et al.

The presence of *UAS-spo* transgenes	Number of 3rd instar of *UAS-dicer/+; phm-GAL4#22/UAS-spok-IR*
+	41 (34)
-	0 (57)
