# Supplementary material for: The Drosophila Zinc Finger Transcription Factor Ouija Board Controls Ecdysteroid Biosynthesis through Specific Regulation of spookier
Source: PLoS Genet. 2015 Dec 10;11(12):e1005712. doi: 10.1371/journal.pgen.1005712 (PMC4684333; doi:10.1371/journal.pgen.1005712)
Supplement: S3 Table — Small letters indicate the restriction enzyme recognition sequences. Under lines indicate the transversion mutation sequences. Asterisks indicate 5´ biotinylation. (PDF) [file pgen.1005712.s003.pdf]

S3 Table.

Komura-Kawa et al.

Purpose	Name of primers	Sequence (5´>3´)
Cloning of *ouib* CDS	CG11762-CDS-Fwd	ATGCTGAACATCGTGTGTAGAGTCTGC
	CG11762-CDS-Rev	TCATGACTTCTGCTTCTTGACATTTTGGA
qRT-PCR primers for amplifying *ouib*	CG11762-qRT-PCR-Fwd	GAGGCGAACGACCATTTGGC
	CG11762-qRT-PCR-Rev	CATACCGCTTTTCGCAGAAGCG
qRT-PCR primers for amplifying *E75A*	E75A-qPCR-FW	CCGGAATTCAGGAGCCTGGTAGCA
	E75A-qPCR-RV	TGGTGCTGACGGCACTCGATTTGC
Vector construction for CRISPR/Cas9 system	CG11762_CRISPR_oligo1_s	cttcGAGCCTGAATCTCTTCGATT
	CG11762_CRISPR_oligo1_as	aaacAATCGAAGAGATTCAGGCTC
	CG11762_CRISPR_oligo2_s	cttcGTGGATTTGGACGATGTTCC
	CG11762_CRISPR_oligo2_as	aaacGGAACATCGTCCAAATCCAC
Amplifying the target sites of CRISPR/Cas9 system	CRISPR_11-1_check_F	AAGTTTTAATATTATAAGCAGTATCTGTTTTATCAGACTGCTC
	CRISPR_11-1_check_R	CCCGACGAAGAGTCGTCCTC
	CRISPR_11-2_check_F	ATCACCATACAATTGCCTTTCCATCAGTC
	CRISPR_11-2_check_R	TCTTCAATCAGTACCTGAAAAGTTTCTTCAACC
Vector construction to overexpress *FLAG-ouib*	CG11762_Fwd_for FLAG	gaattcATGGATTACAAGGATGATGATGATAAGATGCTGAACATCGTGTGTAGAGTCTGC
	CG11762_Rev_for FLAG	tctagaTTATGACTTCTGCTTCTTGACATTTTGGAG
Vector construction to overexpress *FLAG-M1BP*	M1BP-FLAG_F	GAATTCATGGATTACAAGGATGATGATGATAAGATGTCGAAATCGGCGCTGAAACAC
	M1BP-FLAG_R	TCTAGATTAGATGTGCAGGCTGTCCTCCTC

~1.45 kb *spok* promoter-GFP construct	1.45spok-p_F	CGGTGGAAGGTCCTGACCTTTAG
	1.45spok-p_R	TTTCAGCCTTAGTAAATAGTTCTCAACATAC
300 b *spok* promoter-GFP construct	300spok-p_F	TCTTATATACAAAAACCCTTTCCA
	300spok-p_R	ATCTAGACGTACAGATGGGCAGA
Luciferase vector construction	Dm_spok_181-32_F	gagctcTTTGGTATAGTTATAAGCTTTATTATTTAGGCACCATCTTAAAGTTTTAAG
	Dm_spok_171-32_F	gagctcTTATAAGCTTTATTATTTAGGCACCATC
	Dm_spok_161-32_F	gagctcTATTATTTAGGCACCATCTTAAAGTTTTAAG
	Dm_spok_151-32_F	gagctcGCACCATCTTAAAGTTTTAAGACTAC
	Dm_spok_141-32_F	gagctcAAAGTTTTAAGACTACAATAAAATTACTTCTC
	Dm_spok_131-32_F	gagctcGACTACAATAAAATTACTTCTCTTTTTTATAGAATTTATTTTAAATATGCGTTTGAC
	Dm_spok_331-32_R	agatctCTTATATACAAAAACCCTTTCCAACAACAATAAAATTTCATAGG
Mutated luciferase vector construction	spok_all_ver_F	CTAGGGCGGCGGCTGCACCATCTTAAAGTTTTAAGAC
	spok_all_R	TATAACTATACCAAACAAGGC
	spok_1_F	TTTATTATTTAGGCACCATCTTAAAG
	spok_1_ver_R	TAGTATAACTATACCAAACAAGGC
	spok_2_F	ATTATTTAGGCACCATCTTAAAG
	spok_2_ver_R	CCCGCTTATAACTATACCAAACAAGG
	spok_3_F	ATTTAGGCACCATCTTAAAG
	spok_3_ver_R	CCGAAAGCTTATAACTATACCAAACAAG
	spok_4_F	TAGGCACCATCTTAAAGTTTTAAG
	spok_4_ver_R	CCGAATAAAGCTTATAACTATACCAAACAAG
	spok_5_F	GCACCATCTTAAAGTTTTAAGAC
	spok_5_ver_R	AGCAATAATAAAGCTTATAACTATACCAAAC

GFP vector construction	spok_300bp_EcoRI_F	gaattcAATCTAGACGTACAGATGGG
	spok_300bp_XhoI_R	ctcgagCTTATATACAAAAACCCTTTCCAAC
ABCD assay	spok_WT_s2_5biotin	*TTTGGTATAGTTATAAGCTTTATTATTTAGGCACCATCTTAAAGT
	spok_WT_as2	ACTTTAAGATGGTGCCTAAATAATAAAGCTTATAACTATACCAAA
	spok_mutv_s_5bio	*TTTGGTATAGTTATACTAGGGCGGCGGGCTGCACCATCTTAAAGT
	spok_mutv_as	ACTTTAAGATGGTGCAGCCCGCCGCCCTAGTATAACTATACCAAA
	M1BP_wt_l_s	*TCGATACTCCGCACCCAGTGTGACCGTCGAGCGCATGGCATAAA
	M1BP_wt_l_as	TTTATGCCATGCGCTCGACGGTCACACTGGGTGCGGAGTATCGA
EMSA	spok_WT_s2	TTTGGTATAGTTATAAGCTTTATTATTTAGGCACCATCTTAAAGT
	spok_WT_as2	ACTTTAAGATGGTGCCTAAATAATAAAGCTTATAACTATACCAAA
	spok_mutv_s	TTTGGTATAGTTATACTAGGGCGGCGGGCTGCACCATCTTAAAGT
	spok_mutv_as	ACTTTAAGATGGTGCAGCCCGCCGCCCTAGTATAACTATACCAAA
	M1BP_wt_l_s	TCGATACTCCGCACCCAGTGTGACCGTCGAGCGCATGGCATAAA
	M1BP_wt_l_as	TTTATGCCATGCGCTCGACGGTCACACTGGGTGCGGAGTATCGA
