# Supplementary figures and images for: The Drosophila Zinc Finger Transcription Factor Ouija Board Controls Ecdysteroid Biosynthesis through Specific Regulation of spookier
Source: PLoS Genet. 2015 Dec 10;11(12):e1005712. doi: 10.1371/journal.pgen.1005712 (PMC4684333; doi:10.1371/journal.pgen.1005712)

**S1 Fig.**

**Komura-Kawa et al.**

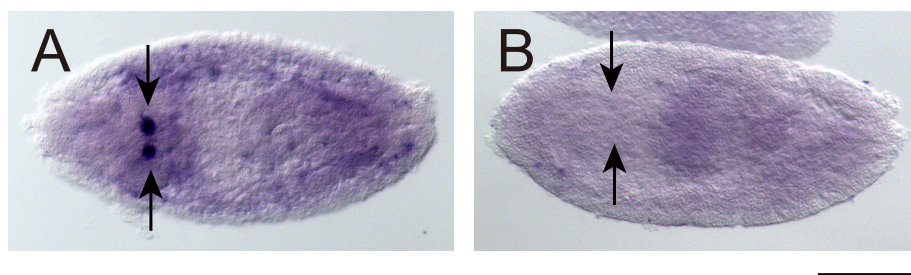

Supplement: S1 Fig — Dorsal views are shown. (A) Signals with antisense probe. (B) Signals with sense probe. Arrows indicate positions of the PG primordia. Scale bar: 100 μm. (PDF) [file pgen.1005712.s004.pdf]

**S2 Fig.**

**Komura-Kawa et al.**

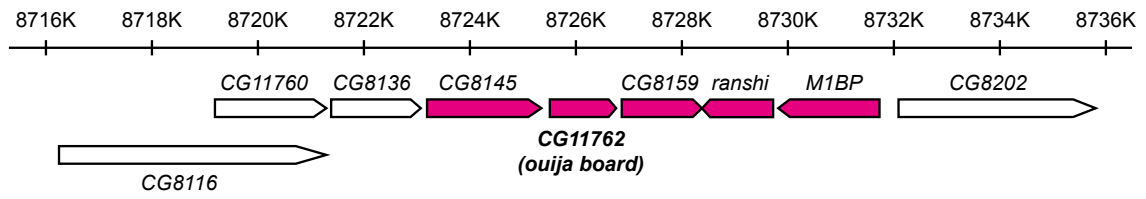

Supplement: S2 Fig — The data are derived from the FlyBase GBrowse website (http://flybase.org/cgi-bin/gbrowse2/dmel/?Search=1;name=FBgn0037618). Numbers indicate the nucleotide positions at the 85A9 cytological position of the chromosome 3R scaffold. Boxed arrows represent gene spans and their directions. The 5 ZAD-ZNF genes are colored by magenta. (PDF) [file pgen.1005712.s005.pdf]

**S3 Fig.**

**Komura-Kawa et al.**

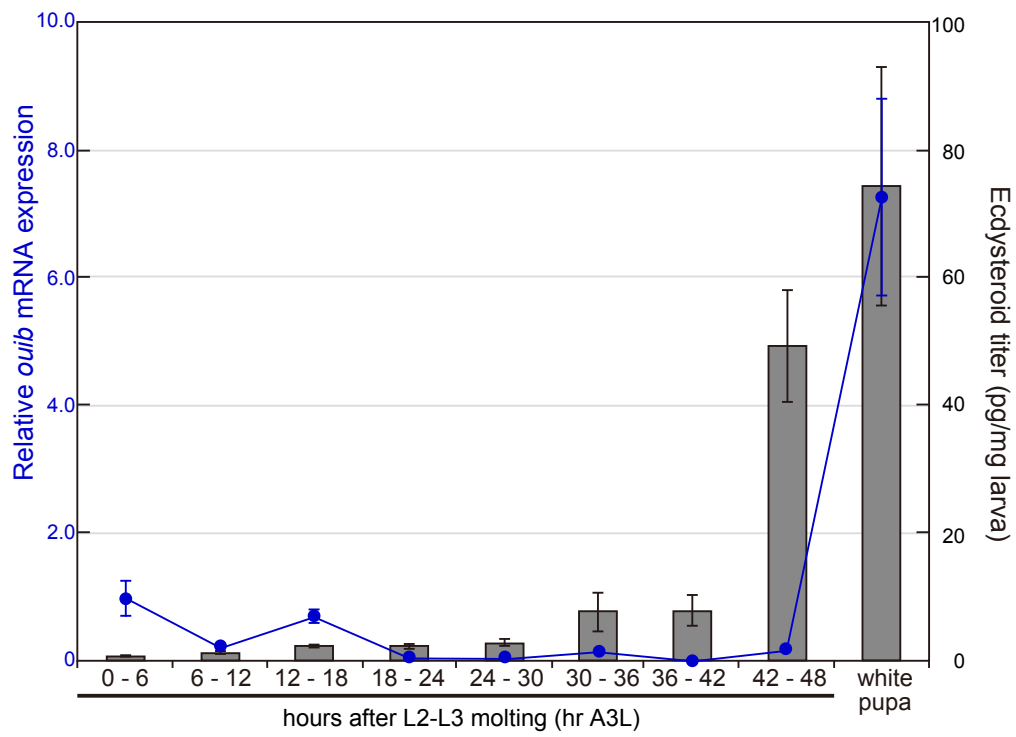

Supplement: S3 Fig — ouib expression and ecdysteroid levels in w 1118 during the 3rd instar stage measured by qRT-PCR (N = 3) and ELISA (N = 4). The blue line indicates the relative expression level of ouib, normalized to the level of 0–6 hours after L2-L3 molting (0–6 hr A3L). Error bars indicate the s. e. m. (PDF) [file pgen.1005712.s006.pdf]

S4 Fig.

Komura-Kawa et al.

A

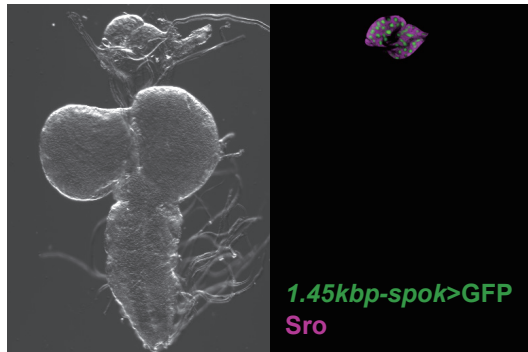

B

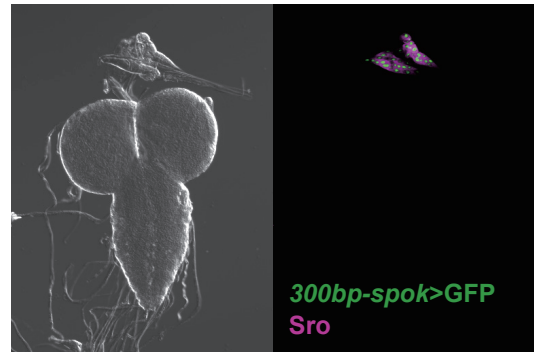

Supplement: S4 Fig — (A, B) Phase-contrast (left) and fluorescence (right) images of the 108 hours AEL 3rd instar larval brain-ring gland complexes with spok>GFP construct. The PG cells were immunostained with anti-Sro antibody (magenta). The spok>GFP constructs contain 1.45 kbp (A) and 300 bp (B) enhancer regiosn of spok, respectively. Scale bar: 100 μm. (PDF) [file pgen.1005712.s007.pdf]

S5 Fig.

Komura-Kawa et al.

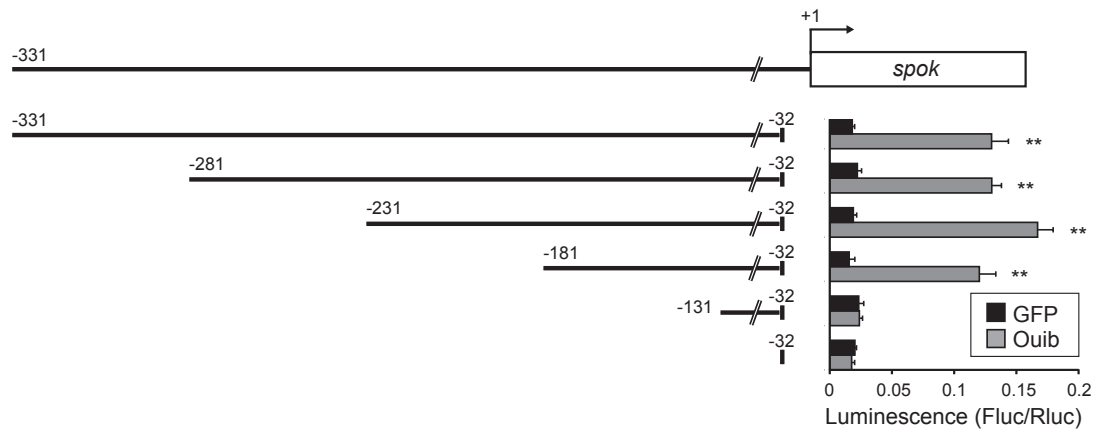

Supplement: S5 Fig — Numbers indicate the distance from the translation initiation site (+1) of spok, and white box represents the coding region of spok. Luc reporter activities of progressive deletion constructs are shown in right. Bars and error bars represent the average and the s. e. m., respectively, of three independent experiments. **; P<0.01 by Student’s t-test. (PDF) [file pgen.1005712.s008.pdf]

S6 Fig.

Komura-Kawa et al.

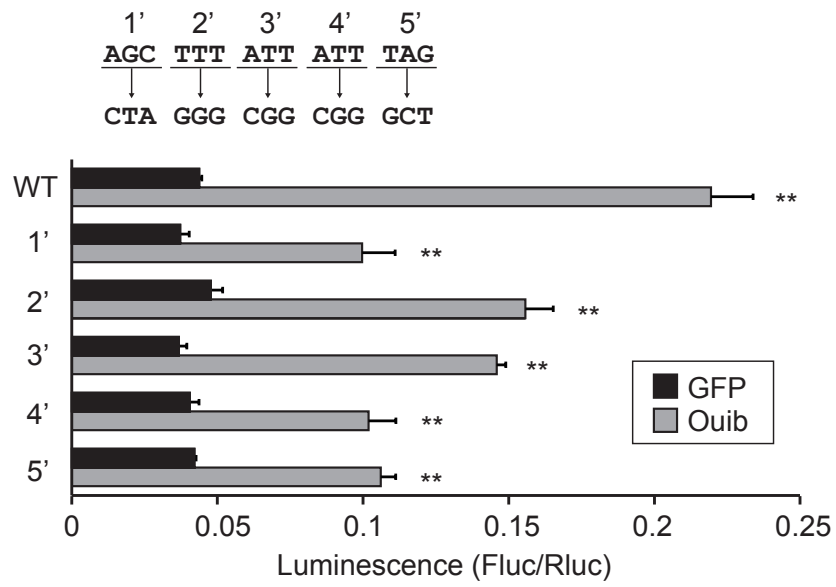

Supplement: S6 Fig — The introduced transversion mutations in the 1’, 2’, 3’, 4’ and 5’ constructs are shown in the top. The GFP expression plasmid was used as a negative control. Bars and error bars represent the average and the s. e. m., respectively, of three independent experiments. **; P<0.01 by Student’s t-test. (PDF) [file pgen.1005712.s009.pdf]

**S8 Fig.**

**Komura-Kawa et al.**

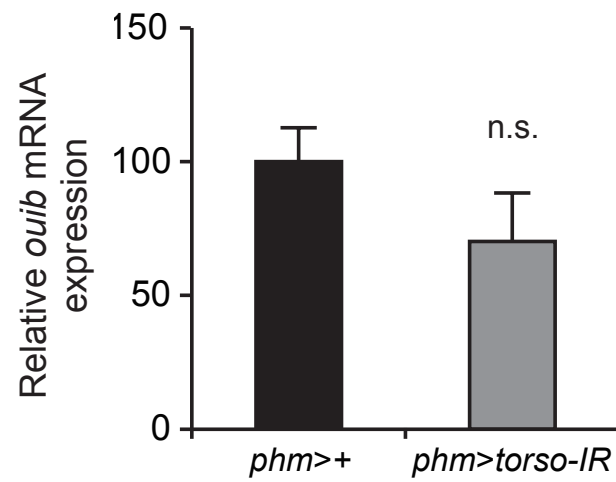

Supplement: S8 Fig — Amounts of ouib mRNAs were measured by qRT-PCR. phm>+ and phm>torso-IR indicate w 1118 ; +/+; phm-GAL4#22/+ and w 1118 ; UAS-torso-IR/+; phm-GAL4#22/+, respectively. RNA samples were collected 140 hours after egg laying. Bars and error bars represent the average and the s. e. m., respectively, of three biological replicates. n.s. means P>0.05 by Student’s t-test. (PDF) [file pgen.1005712.s011.pdf]

**S10 Fig.**

**Komura-Kawa et al.**

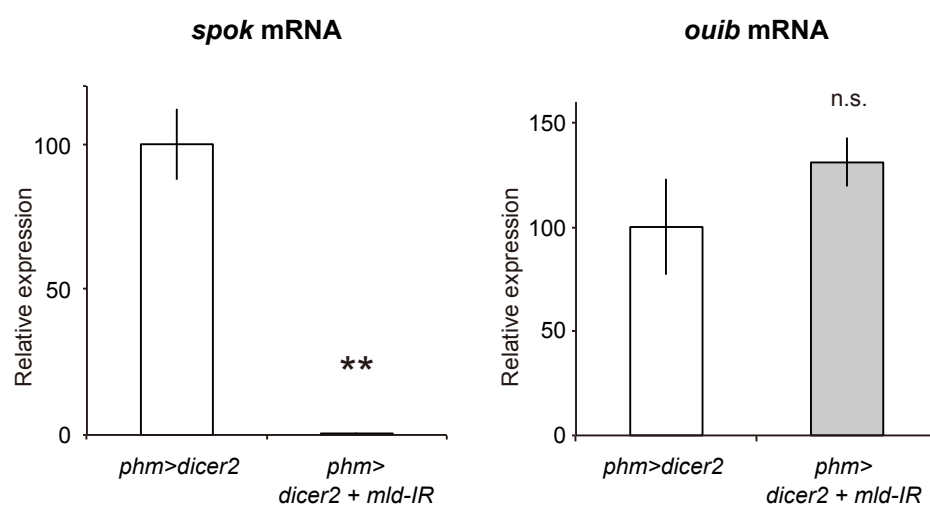

Supplement: S10 Fig — Amounts of spok and ouib mRNAs were measured by qRT-PCR. phm>dicer2 and phm>dicer2+mld-IR indicate w 1118 ; UAS-dicer2/+; phm-GAL4#22/+ and w 1118 ; UAS-dicer2/UAS-mld-IR; phm-GAL4#22/+, respectively. RNA samples were collected 36 hours after egg laying. Bars and error bars represent the average and the s. e. m., respectively, of three biological replicates. ** and n.s. mean P<0.01 and P>0.05 by Student’s t-test, respectively. (PDF) [file pgen.1005712.s013.pdf]
