# Supplementary material for: The Drosophila Zinc Finger Transcription Factor Ouija Board Controls Ecdysteroid Biosynthesis through Specific Regulation of spookier
Source: PLoS Genet. 2015 Dec 10;11(12):e1005712. doi: 10.1371/journal.pgen.1005712 (PMC4684333; doi:10.1371/journal.pgen.1005712)
Supplement: S7 Fig — EMBOSS Matcher [54] was used to search for sequences similar to the D. melanogaster Ouib response element (15 bp) within the 1 kb regions upstream of the translation initiation site of the spok loci from 12 Drosophilidae species. Numbers before and after nucleotide sequences indicate the distance from the translation initiation site of spok. Parentheses indicate numbers of identical matches to D. melanogaster Ouib response element. “S” and “D” indicate the subgenera Sophophora and Drosophila, respectively. spok genes are DG27210 (D. simulans #1), GD27133 (D. simulans #2), GD28291 (D. simulans #3), GM22791 (D. sechellia), GG16659 (D. erecta), GE19452 (D. yakuba), GF20000 (D. ananassae), GA31537 (D. pseudoobscura), GL21970 (D. persimilis), GK19177 (D. willistoni), GI23968 (D. mojavensis), GH21174 (D. grimshawi) and GJ26360 (D. virilis). Note that a BLAST search using D. melanogaster Spok protein sequence as a query hit 3 D. simulans spok candidate genes. Also note that the BLAST search hit only one spo/spok family gene in D. grimshawi genome and thus it is not faithfully judged if GH21174 is orthologous to spo or spok. (PDF) [file pgen.1005712.s010.pdf]

S7 Fig.

Komura-Kawa et al.

melanogaster (S)	-166	AGCTTTATTATTTAG	-152	
simulans-1 (S)	-451	AGCTTTCGACTTTCG	-437	(10/15)
simulans-2 (S)	-906	AGCTTCTATATTTGG	-892	(11/15)
simulans-3 (S)	-561	AGCATTAATCACTTG	-547	(9/15)
sechellia (S)	-451	AGCTTTCGACTTTCG	-437	(10/15)
erecta (S)	-203	AGCTTTATGAATTAG	-189	(13/15)
yakuba (S)	-217	AGCTTTATTATTTAG	-203	(15/15)
ananassae (S)	-97	AGCTTTTATAGTTAG	-83	(11/15)
pseudoobscura (S)	-114	AGCTTTGTCGCTGAG	-100	(10/15)
persimilis (S)	-114	AGCTTTGTCGCTGAG	-100	(10/15)
willistoni (S)	-395	CCCTTTAAAATTTAT	-381	(10/15)
mojavensis (D)	-721	AGCTTTGTTAAATTT	-707	(10/15)
grimshawi (D)	-846	AACATTATAATTGCG	-832	(8/15)
virilis (D)	-419	AATATTTTTATTTAG	-405	(10/15)
