# Supplementary material for: The Drosophila Zinc Finger Transcription Factor Ouija Board Controls Ecdysteroid Biosynthesis through Specific Regulation of spookier
Source: PLoS Genet. 2015 Dec 10;11(12):e1005712. doi: 10.1371/journal.pgen.1005712 (PMC4684333; doi:10.1371/journal.pgen.1005712)
Supplement: S9 Fig — EMBOSS Matcher [54] was used to search for sequences similar to D. melanogaster Ouib response element (15 bp) within the putative enhancer/promoter regions of D. melanogaster ecdysteroidogenic enzyme genes. Numbers before and after nucleotide sequences indicate the distance from the translation initiation site of each gene. Parentheses indicate numbers of identical matches to D. melanogaster Ouib response element. Except for phm, a enhancer/promoter region was defined as a genomic region between the translation initiation site of each ecdysteroidogenic enzyme gene and the 3´ end of a gene next to the enzymatic gene. A phm enhancer/promoter is a -500 to -1 region as previously characterized [31]. (PDF) [file pgen.1005712.s012.pdf]

S9 Fig.

Komura-Kawa et al.

spok	-166	AGCTTTATTATTTAG	-152	
nobo	-72	TGCTTTTCAATTCAG	-58	(10/15)
nvd	-283	AGCTTTATTGCTCAG	-269	(12/15)
spo	-229	ATCCTGATTATTCAT	-215	(10/15)
sro	-90	TGCATTTTTTTTTGT	-76	(9/15)
Cyp6t3	-35	AGTTATAATATTCTA	-21	(9/15)
phm	-417	CCCTGTATGCTATAG	-403	(9/15)
dib-1	-205	GGCTCTCTTAATTTT	-191	(9/15)
dib-2	-238	ACCTTTCTTTTTAAG	-224	(11/15)
sad	-791	GGCTTAACTAATTAA	-777	(9/15)
